# Comprehensive analysis of N^6^-methyladenosine regulators with the tumor immune landscape and correlation between the insulin-like growth factor 2 mRNA-binding protein 3 and programmed death ligand 1 in bladder cancer

**DOI:** 10.1186/s12935-022-02456-7

**Published:** 2022-02-11

**Authors:** Jianfeng Cui, Yaofeng Zhu, Xiaochen Liu, Wenfu Wang, Xuewen Jiang, Yangyang Xia, Guanwen Zhou, Shouzhen Chen, Benkang Shi

**Affiliations:** 1 Department of Urology, Qilu Hospital, Cheeloo College of Medicine, Shandong University, Jinan, 250012 Shandong China; 2 Key Laboratory of Urinary Precision Diagnosis and Treatment in Universities of Shandong, Jinan, 250012 Shandong China; 3 The Key Laboratory of Experimental Teratology, Ministry of Education and Department of Molecular Medicine and Genetics, School of Basic Medical Sciences, Shandong University, Jinan, 250012 Shandong China

**Keywords:** m^6^A, Immune landscape, Bladder cancer, IGF2BP3, PD-L1

## Abstract

**Background:**

N^6^-methyladenosine (m^6^A) is one of the most abundant post-transcriptional modifications of RNA. However, there is limited information about the potential roles of m^6^A regulators in tumor immunity. Therefore, in this study, we aimed to testify the functions of m^6^A regulators in bladder cancer as well as their association with the tumor immune landscape.

**Methods:**

We reported the variation and expression levels of m6A regulators in the TCGA database and GTEx database of bladder cancer. Clusters, risk score patterns, and nomograms were constructed to evaluate the function and prognostic value of m^6^A regulators. Furthermore, we constructed nomogram to evaluate the prognosis of the individual patients. The correlation between insulin-like growth factor 2 mRNA-binding protein 3 (*IGF2BP3*) and programmed cell death ligand 1 (*PD-L1*) was evaluated both in vitro and in vivo.

**Results:**

We found that the tumor grade and DNA damage pathways were strongly correlated with distinct clusters. Furthermore, two risk score groups with six m^6^A regulators were identified using the least absolute shrinkage and selection operator (LASSO) and multivariable Cox regression analysis, which could be regarded as independent prognostic markers in patients with bladder cancer. The risk score pattern was linked to the tumor immune landscape, indicating a correlation between immune checkpoints and m^6^A regulators. Moreover, an m^6^A regulator, *IGF2BP3*, was found to be highly expressed in the tumor samples, regulating both the total and membrane-bound *PD-L1* expression levels.

**Conclusions:**

The results of this study revealed that the m^6^A clusters and patterns play crucial roles in the regulation of tumor immunity, which may be used to develop comprehensive treatment strategies for the management of bladder cancer.

**Supplementary Information:**

The online version contains supplementary material available at 10.1186/s12935-022-02456-7.

## Background

Of the 171 post-transcriptional modifications of RNA, including 5-methylcytosine (m^5^C), 7-methylguanosine (m^7^G), m^1^G, m^2^G, and m^6^G, N^1^-methyladenosine (m^1^A) and N^6^-methyladenosine (m^6^A) have been identified in living organisms [1]. m^6^A is one of the most prominent and abundant internal modifications in eukaryotic mRNA and long noncoding RNA (lncRNA), accounting for 0.2–0.4% of the total adenosine residues and half of the total ribonucleotides in mammalian RNA [2–4]. Similar to dynamic and reversible epigenetic modifications of genomic DNA and proteins, m^6^A RNA modification is a reversible process in mammalian cells, which may be regulated by three vital factors, namely the methyltransferases, demethylases, and binding proteins, which are also known as writers, erasers, and readers, respectively. They can add, remove, or read an m^6^A site [5, 6]. m^6^A modifications are related to various biological functions, such as RNA splicing, translocation, stability, and translation [7, 8], as well as multiple dysregulated biological processes, including aberrant proliferation, promotion of tumor metastasis, and inhibition of apoptosis [9–12].

Tumor progression is attributed to multiple genetic and epigenetic variations in tumor cells [8]. However, increasing evidence has shown that evading tumor surveillance is a hallmark of tumor development [13]. The immunogenic interaction between the host tissues and the tumors, and the ability of the tumor to evade immune recognition could determine the prognosis of patients [14]. Immunotherapy targeting immunological checkpoints, such as programmed cell death 1 (*PD-1*), programmed cell death 1 ligand 1 (*PD-L1*), and cytotoxic T lymphocyte-associated antigen-4 (*CTLA-4*), has been used as a potential therapeutic strategy for cancers. As cancers are not solely neoplastic cells, they contain the tumor microenvironment (TME), which can be divided into immune and non-immune infiltrates, such as cytotoxic T cells, natural killer cells, dendritic cells (DCs), tumor-associated macrophages (TAMs), endothelial cells, and stromal cells [15]. The TME is a highly complex ecosystem, and various biological behaviors change through direct and indirect interactions with the TME components. Thus, it is important to clarify the immune infiltration at the tumor site and the biomarkers associated with TME, which might help to individually evaluate patients who could benefit from immunotherapies and will broaden our understanding of tumor immunity.

Bladder cancer (BCa) is more common in men than in women, and ranks 4th in incidence and 8th in mortality among the male population, according to the latest published cancer statistics [16]. Approximately 75% of patients with newly diagnosed BCa are non-muscle invasive bladder cancer (NMIBC), and 25% are muscle invasive bladder cancer (MIBC) [17]. Bacillus Calmette-Guérin (BCG), which can activate human innate and adaptive immune responses, intravesical instillation is the current gold standard clinical treatment for NMIBC. Meanwhile, *anti-PD-1/PD-L1* immunotherapy is the hotspot for advanced MIBC, and the response rate of immunotherapies is determined by various conditions, including tumor immunity and cancer cell immunogenicity [18, 19]. Recent studies have revealed an interaction between m^6^A modification and the TME. A study by Jiang et al. revealed that when co-cultured with M2 macrophages, the expression levels of alkB homolog 5 (*ALKBH5*) and the toll-like receptor (*TLR*)-4 increased in ovarian cancer cells, and *TLR4* upregulated *ALKBH5* expression and increased Nanog expression via mRNA demethylation [20]. Moreover, *TNF-α* inhibits the differentiation of mesenchymal stem cells by repressing fat mass and obesity‐associated protein (*FTO*) expression and *FTO*-mediated demethylation of Nanog mRNA levels and decreasing Nanog mRNA expression levels [21]. Zhang et al. integrated gastric cancer samples to establish m^6^A modification patterns and scoring systems, and found that TME characteristics were highly consistent with the patterns, suggesting that m^6^A modification played an insignificant role in tumor immunity in gastric cancer [22].

However, the above studies only investigated one or two m^6^A regulators and did not investigate the connection between m^6^A regulators and tumor immunity. Therefore, in this study, we comprehensively investigated the tumor immune landscape associated with m^6^A regulators, established a set of scoring patterns, and evaluated the prognostic value of this pattern for individual patients.

## Methods

### Dataset source acquisition

All data were obtained from The Cancer Genome Atlas (TCGA)-Urothelial Bladder Carcinoma (BLCA) database (https://portal.gdc.cancer.gov/) and the Genotype Tissue Expression project (GTEx) database (https://gtexportal.org/), including RNA sequencing data (fragments per kilobase of transcript per million fragments sequenced (FPKM) value) of gene expression, copy number variation (CNV), somatic mutation, and clinical information. The GTEx database includes over 10,000 bulk RNA-seq samples representing 53 different tissues (corresponding to 30 organs) obtained from 635 pre-healthy individuals, to link the influence of genetic variants on gene expression levels via quantitative trait loci analysis (eQTL) [23]. The expression levels of normal samples from TCGA and GTEx databases were integrated and used for comparison with tumor samples. The Cancer Cell Line Encyclopedia (CCLE) database was used to evaluate the expression levels of the m^6^A regulator in several cell types, and the correlation with *PD-L1* at the bladder cancer cell level. The protein levels of m^6^A regulators were determined using the Human Protein Atlas database (https://www.proteinatlas.org/). The Oncomine database was used to determine the insulin-like growth factor 2 mRNA-binding protein 3 (*IGF2BP3*) expression levels in several cancer types (https://www.oncomine.org/). The gene expression profiling interactive analysis (GEPIA) database was used to evaluate the prognostic value of *IGF2BP3* (http://gepia.cancer-pku.cn/). The workflow of this study is shown in Additional file 1: Fig. S1.

### Selection of m^6^A regulators

A total of 24 m^6^A RNA methylation regulators were extracted from the database according to the relevant m^6^A studies [22, 24], including nine writers [*METTL3*, *METTL14*, *METTL16*, RNA-binding motif protein (*RBM15*)*-15*, *RBM15B*, WT1-associated protein (*WTAP*), *KIAA1429*, Cbl proto-oncogene like 1(*CBLL1*)*,* and zinc finger CCCH-type containing 13 (*ZC3H13*)], two erasers (*ALKBH5* and *FTO*), and 13 readers [YTH domain containing *YTHDC1*, *YTHDC2*, *YTHDF1*, *YTHDF2*, *YTHDF3*, *IGF2BP1*, *IGF2BP2*, *IGF2BP3*, heterogeneous nuclear ribonucleoprotein A2/B1 (*HNRNPA2B1*), heterogeneous nuclear ribonucleoprotein C (*HNRNPC*), *FMR1*, leucine rich pentatricopeptide repeat containing (*LRPPRC*), and ELAV-like RNA binding protein 1 (*ELAVL1*)]. The extracted information was used for further analyses.

### Consensus clustering analysis

Unsupervised clustering of BCa samples was conducted to identify different m^6^A regulatory patterns based on their mRNA expression levels and classify the patients for further analysis. The “ConsensuClusterPlus” R package was used to perform the consensus clustering algorithm, which could determine the number of clusters and assess their stability according to the m^6^A regulator expression levels [25]. “k” was used to represent the number of subgroups. Principal component analysis (PCA) was conducted to verify the grouping results, and the set of protein in PCA is the total of 24 m^6^A RNA methylation regulators.

### Identification of differentially expressed genes (DEGs) and construction of the protein–protein interaction (PPI) network

The “limma” R package was applied to identify the differentially m^6^A expressed genes between normal and tumor samples [26]. The significance criteria for determining DEGs was set as the P value < 0.05, and |log2FC|> 1. The Search Tool for the Retrieval of Interacting Genes/Proteins (STRING) database and Cytoscape software was used to retrieve and construct a PPI network of the m^6^A regulator network.

### Gene set variation analysis (GSVA)

To investigate the changes in pathway activity between different groups, GSVA was performed using the “GSVA” R packages. GSVA is a non-parametric, unsupervised method for estimating the variation in gene set enrichment and biological process activity of samples from an expression dataset [27]. In the present study, the gene sets of “c2.cp.kegg.v7.1.symbols” were downloaded from the Molecular Signatures Database (MsigDB) for running the GSVA analysis. We defined an adjusted P value < 0.05, and |log2FC|≥ 0.08, as statistically significant.

### Estimation of immune signatures, TME cell infiltration level and tumor purity in BCa

The single-sample gene-set enrichment analysis (ssGSEA) algorithm was used to quantify the relative abundance of immune signatures in the TME. The enrichment levels of 29 immune signatures were quantified for each sample. The gene sets representing each immune signature are shown in Additional file 1: Table S1, including B cells, Th2 cells, NK cells MHC class I cells, CD8 + T cells, and so on [28]. The “sparcl” R package was employed to divide the samples into three groups, including immunity high, medium and low for further analyses. ESTIMATE was used to evaluate TME cell infiltration level (including immune and stromal scores) and tumor purity for each sample [29].

### Cox regression analyses

Univariable and multivariable Cox regression analyses were used to assess the prognostic value of m^6^A regulators, and hazard ratios (HRs) > 1 or < 1 were regarded as risk and protective genes, respectively. The least absolute shrinkage and selection operator (LASSO) Cox regression algorithm was used to construct the optimal prognostic model out of the m^6^A regulators using the “glmnet” package in R. The LASSO analysis performed predictor selection, minimized over-fitting, selected genes to reduce bias, and developed the best survival-associated risk pattern [30]. After testing for collinearity, the sum of the Cox coefficient and gene values were calculated using the risk score based on the following formula:

$$Risk\,score=\sum (coefficient\times expression\,of\,signature\,gene)$$

Each patient was assigned a risk score based on integrative m^6^A regulator expression patterns. Tree-fold cross-validation and 1000 iterations were conducted to reduce the potential instability of the results.

### The correlation between the expression levels of m^6^A regulators and mutation with immune cell infiltration

To explore the relationship between m^6^A regulators involved in the risk score pattern and the infiltrating immune cells, we utilized the Tumor IMmune Estimation Resource (TIMER) web tool (https://cistrome.shinyapps.io/timer/) to calculate the correlation coefficients between m^6^A regulator expression and mutations with infiltrated immune cells including B cells, CD4 + T cells, CD8 + T cells, macrophages, neutrophils, and dendritic cells [31, 32].

### Construction and validation of a predictive nomogram

Based on the results of the multivariable Cox regression model, a nomogram based on independent prognostic factors was constructed to predict 3- and 5-year OS. The nomogram provides a graphic representation linking individual patient factors to predict the survival probability of BCa patients [33]. In addition, a bootstrapped resample with 1000 iterations was applied to verify the accuracy of the nomogram. Furthermore, the performance of the prognostic models was evaluated by receiver operating characteristic (ROC) analyses, and the concordance index (c-index) was measured to quantify the nomogram discrimination. A scale of 1.0, represents perfect predictions, and 0.5, the equivalent of a coin toss. The calibration of the model was assessed by comparing the observed survival with the predicted survival by plotting a calibration curve. The 45° line indicates a perfect calibration. Any deviation above or below the 45° line indicates underprediction or overprediction, respectively. Due to the limited conditions, no extra BCa cohort could be used as an externally validated database to evaluate the efficacy of model validation and prediction. Thus, only internal validation was conducted to evaluate the nomogram model.

### Tissue specimens

This study was approved by the Medical Ethics Committee of the Shandong University School of Clinical Medicine. Twenty human BCa tissues were collected at the Qilu Hospital of Shandong University. Informed consent was obtained from all patients.

### Cell cultures and manipulation

T24, 5637, and UMUC3 cell lines were purchased from the American Type Culture Collection (ATCC). T24 and 5637 cells were cultured in the Roswell Park Memorial Institute (RPMI)-1640 medium (Gibco; 11875093). UMUC3 cells were cultured in Dulbecco’s modified Eagle’s medium (DMEM; Gibco; 11995065). All media were supplemented with 10% fetal bovine serum (Gibco; 10099-141C). The cells were incubated at 37 °C in a humidified atmosphere with 5% CO_2_.

Stable *IGF2BP3* knockdown, overexpression cell lines, and their controls were generated as described previously [34]. Lentiviruses were purchased from GeneChem Inc. (Shanghai, China).

### Western blotting and antibodies

Western blotting was performed as previously described [34]. The primary antibodies included *anti-IGF2BP3* (Abcam; ab177477), *anti-PD-L1* (Proteintech; 28076-1-AP), and *anti-GAPDH* (Proteintech; 60004-1-Ig).

### RNA extraction and reverse transcription-polymerase chain reaction (RT-PCR)

Extraction of total RNA and RT-PCR were performed as previously described [34]. Primers used were purchased from Sangon Biotech (Shanghai, China), and primer sequences are shown in Additional file 1: Table S2.

### Flow cytometric assay

Flow cytometric assays were performed as previously described [35]. Briefly, single-cell suspensions were freshly prepared from the inidicated cells. Cells were washed once with PBS and stained with anti-PD-L1 antibody (Biolegend, 329705) for 30 min. The samples were analyzed on a FACSCanto II (BD Bioscience, USA) using FlowJo 7.6.5.

### Statistical analysis

All analyses were conducted using the R v.4.0.0 and SPSS v.20.0 software. The “RCircos” R package was used to plot the CNV landscape of m^6^A regulators. The somatic mutation landscape was assessed using the “maftools” R package to plot the mutation summary, waterfall, and gene cloud figures. The correlations among different m^6^A regulators were computed by “corrplot” R package. “Survival” R package was adopted to analyze Kaplan–Meier curve analysis. The “forestplot” R package was conducted to visualize the univariable and multivariable prognostic analysis for risk score. The specificity and sensitivity of risk score were assessed using the ROC curve and the area under the curve (AUC), which were quantified by the “pROC” R package. Data from the two groups were evaluated using a two-tailed unpaired Student’s t-test. Categorical data were analyzed using the chi-square test. The correlation between continuous variables was assessed using the Spearman’s correlation analysis. Survival analysis was performed using log-rank test. Statistical significance was set at P < 0.05.

## Results

### Landscape of somatic mutation and CNV mutation of m^6^A regulators in BCa

**Fig. 1:**
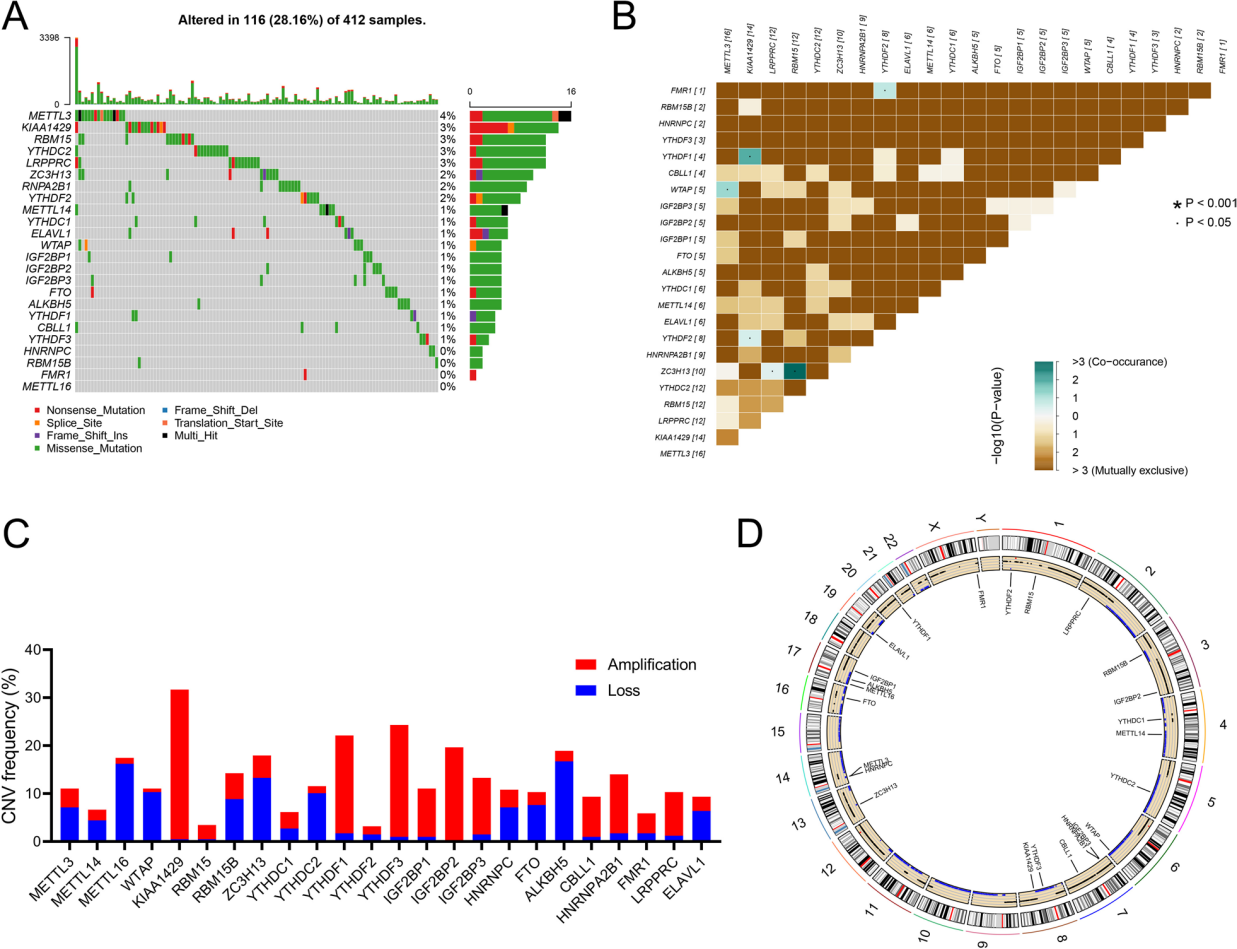
Landscape of the somatic and copy number variation (CNV) mutations of m^6^A regulators in bladder cancer (BCa). **A** The mutation profile of 24 m^6^A regulators in patients with BCa. The upper barplot indicates the tumor mutational burden (TMB) of individual patients, and the number on the right shows the mutation frequency in each regulator. **B** The m^6^A somatic mutation co-occurrence and mutually exclusion analyses of 24 m^6^A regulators. Co-occurrence to mutual exclusion from green to brown. **C** The CNV variation frequency of m^6^A regulators in BCa. The blue column represents the deletion frequency, and the red column represents the amplification frequency. **D** The location of CNV alteration of m^6^A regulators on different chromosomes. ·P < 0.05; *P < 0.001

A total of 24 m^6^A regulators in BCa were used in the present study. We first clarified the incidence of CNV and somatic mutations in m^6^A regulators in BCa. Among the 412 samples, 116 (28.2%) experienced somatic mutations in m^6^A regulators (Fig. 1A). *METTL3* showed the highest mutation frequency among the m^6^A regulators (approximately 4%), while two writers (*RBM15B* and *METTL16*) and two readers (*HNRNPC* and *FMR*) did not exhibit any somatic mutations in BCa. Correlation analyses revealed that most m^6^A somatic mutations did not exhibit a co-occurrence relationship, except for *FMR1* and *YTHDF2*, *YTHDF1* and *KIAA1429*, *WTAP* and *METTL3*, *ZC3H13*, and *RBM15* (Fig. 1B). Next, we summarized the CNV mutation frequency among the m^6^A regulators, and *KIAA1429*, *YTHDF1*, *YTHDF3*, and *IGF2BP2* had a widespread frequency of CNV amplification, while METTL16 and ALKBH5 showed high CNV deletion frequency (Fig. 1C). We also explored the CNV mutation in normal tissues, and only 7 m^6^A regulators had a CNV mutation burden, with an extremely low frequency (Additional file 1: Fig. S2). The location of the CNV mutation in m^6^A regulators on different chromosomes is shown in Fig. 1D.

### Profiles of mRNA and protein expression level of m^6^A methylation regulators in BCa

**Fig. 2:**
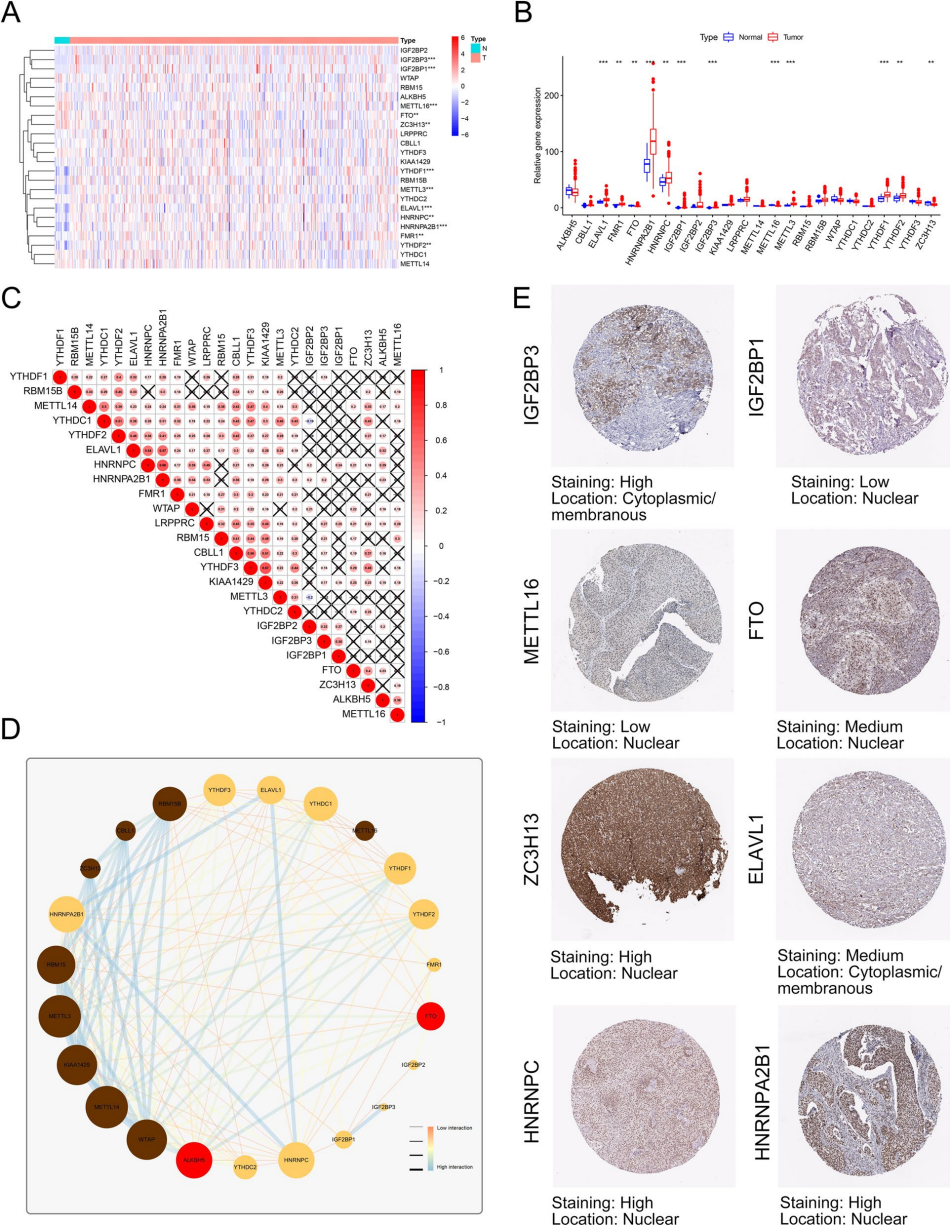
Profiles of expression levels of 24 m^6^A methylation regulators in BCa and adjacent normal tissues. **A** Heatmap of m^6^A RNA expression levels in BCa and normal tissues from The Cancer Genome Atlas (TCGA) and Genotype Tissue Expression project (GTEx) databases. **B** Box plots of m^6^A RNA expression levels of the tumor and normal tissues. **C** Spearman correlation analysis of the m^6^A regulators in BCa. Red dot represents positive correlation and blue dot represents negative correlation. **D** The interaction among m^6^A regulators in BCa. Brown dots represent the writers, red dots represent the erasers, and yellow dots represent the readers. The lines linking regulators show their interactions, while the thickness and color show the correlation strength between the regulators. Low interaction is marked with thin orange lines, while positive correlation is marked with blue thick lines. **E** The protein levels of m^6^A regulators detected by immunohistochemistry staining were from The Human Protein Atlas database

After exploring the mutation of m^6^A regulators, we investigated the mRNA expression levels of m^6^A regulators in normal bladder samples and tumor samples. The GTEx and TCGA datasets were merged for further analysis, with 28 normal samples and 411 tumor samples. As shown in Fig. 2A, B, mRNA expression levels of *CBLL1*, *ELAVL1*, *HNRNPA2B1*, *IGF2BP1*, *IGF2BP2*, *IGF2BP3*, *LRPPRC*, *RBM15*, *RBM15B*, *YTHDF1*, *YTHDF2*, and *YTHDF3* were significantly higher in tumor samples than in healthy samples, while the expression levels of *FTO*, *METTL14*, *METTL16*, *WTAP*, *YTHDC1*, *YTHDC2*, and *ZC3H13* were decreased in tumor samples. Due to the functional similarity or complementation, the comprehensive landscape of m^6^A regulator connections was depicted by Spearman correlation analysis, STRING website, and Cytoscape software, *METTL3* and *YTHDF3* showed the strongest positive correlation, while *METTL3* and *IGF2BP2* showed the strongest negative relevance (Fig. 2C, D). Not only did the m^6^A regulators with similar functions show a significant correlation, but a remarkable interaction was shown among writers, erasers, and readers. Moreover, correlations between writers and erasers were investigated to determine whether tumors with high eraser expression levels exhibited low writer expression levels. The results revealed that tumors with high expression of *CBLL1* and *METTL14* showed a high expression of *FTO*, while the high expression of *CBLL1* and *METTL14* showed low expression of *ALKBH5*. Tumors with high expression of *ZC3H13* and *WTAP* showed high expression of *FTO*. However, *ZC3H13* and *WTAP* did not interfere with *ALKBH5* expression. The remaining writer genes did not affect the eraser genes *ALKBH5* and *FTO* (Additional file 1: Fig. S3A). Immunohistochemistry staining images of m^6^A regulator proteins were retrieved from the Human Protein Atlas, revealing cellular sublocalization and intensity (Fig. 2E and Additional file 1: Fig. S3B). The above results revealed that cross-talk among m^6^A regulators might construct important modification patterns.

### Identification of m^6^A regulators in two subgroups using consensus clustering

**Fig. 3:**
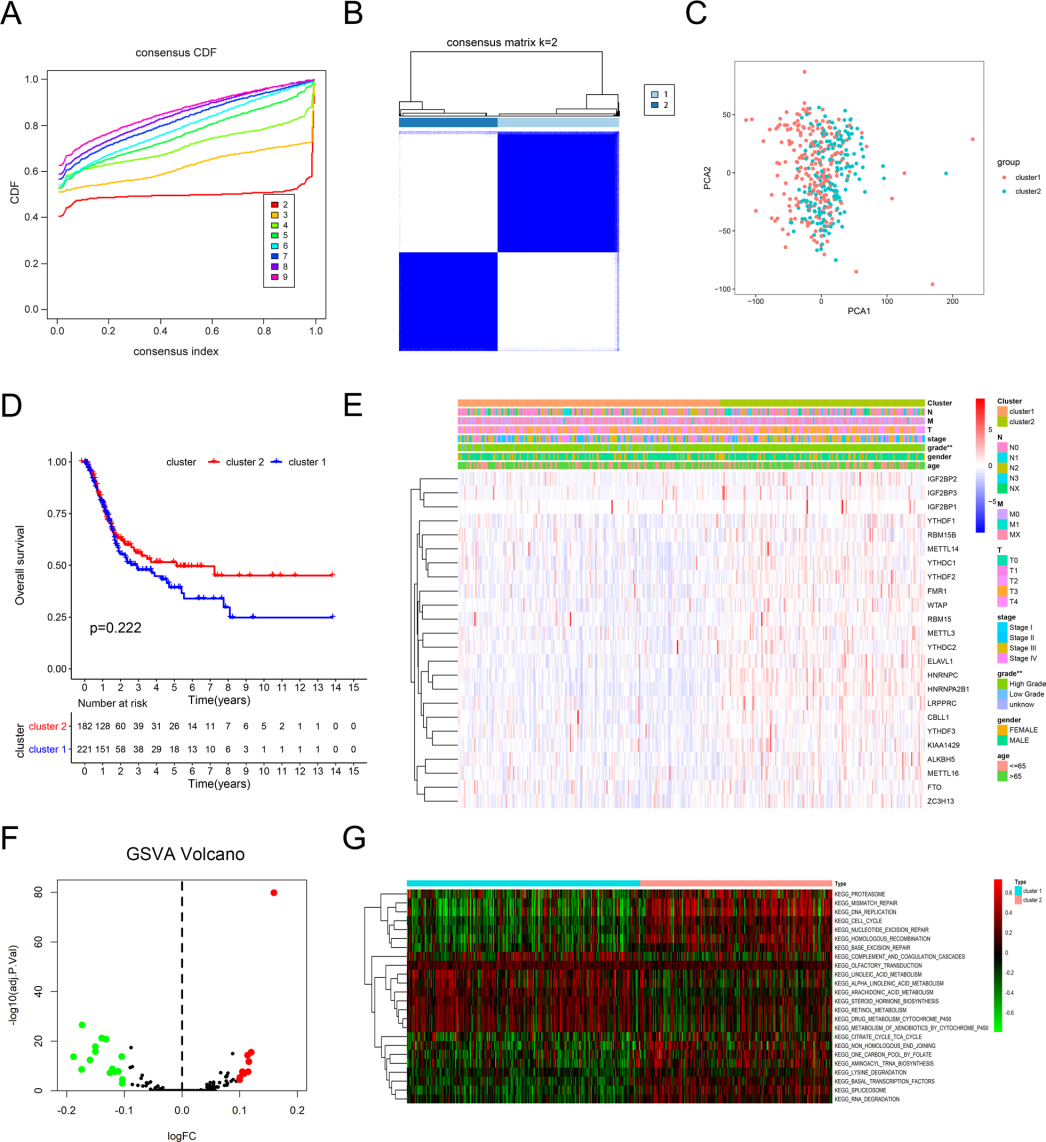
Identification of consensus clusters by m^6^A regulators associated with the clinicopathological characteristic and pathway. **A** Consensus clustering cumulative distribution function (CDF) diagram with a clustering number from k = 2 to k = 9. **B** Consensus clustering matrix for k = 2, displaying the clustering stability using 1000 iterations of hierarchical clustering. **C** Principal component analysis (PCA) for the transcriptome profiles of two consensus clusters. **D** Kaplan–Meier curves for patients with BCa. Patients in cluster 1 were marked with blue, while those in cluster 2 were marked with red. **E** Heatmap and clinicopathologic features of the two clusters classified by the m^6^A regulators consensus expression. The m^6^A cluster, N status, M status, T status, tumor stage, tumor grade, gender, and age were used as patient annotations. Red represents the high expression of m^6^A regulators and blue represents the low expression. **F**–**G** Gene set variation analysis (GSVA) enrichment analysis showed the status of biological pathways between the two clusters. The volcano plot was used to visualize these biological processes. Red dots represents the activated pathways and green dots represents the inhibited pathways, logFC set as 0.1, and P value set as 0.05 (**F**), and the heatmap was used to visualize these biological processes and red represented activated pathways and green represented inhibited pathways (**G**)

The empirical cumulative distribution function was plotted to analyze the optimal k value at which the cluster model achieved maximum stability (Fig. 3A and Additional file 1: Fig. S4A). The results showed that k = 2 gained the most powerful clustering efficacy, and the samples were divided into two subclusters using unsupervised clustering (Fig. 3B and Additional file 1: Fig. S4B-F). PCA analysis was used to judge the classification, and the two clusters could gather together (Fig. 3C). Prognostic analysis for the two clusters did not show a statistically significant difference, but a trend in overall survival (OS) (Fig. 3D). We explored PCA analysis and prognostic analysis for k = 3 and 4, and no significant benefits in OS were found (data not shown). However, the correlation analysis showed that clustering was associated with the tumor grade (Fig. 3E). The m^6^A expression profiles showed that all m^6^A regulators were upregulated in cluster 2, except for IGF2BP1 (Additional file 1: Fig. S4G). GSVA enrichment analysis was performed to explore the biological behaviors of the two clusters. As shown in Fig. 3F–G, cluster 1 presented enrichment pathways associated with metabolism, such as linoleic acid, arachidonic acid, retinol, drug, and xenobiotic metabolism. Cluster 2 was remarkably enriched in DNA damage, including mismatch repair, DNA replication, cell cycle, nucleotide excision repair, and spliceosome.

### Characteristics of risk score pattern based on m^6^A regulators

**Fig. 4:**
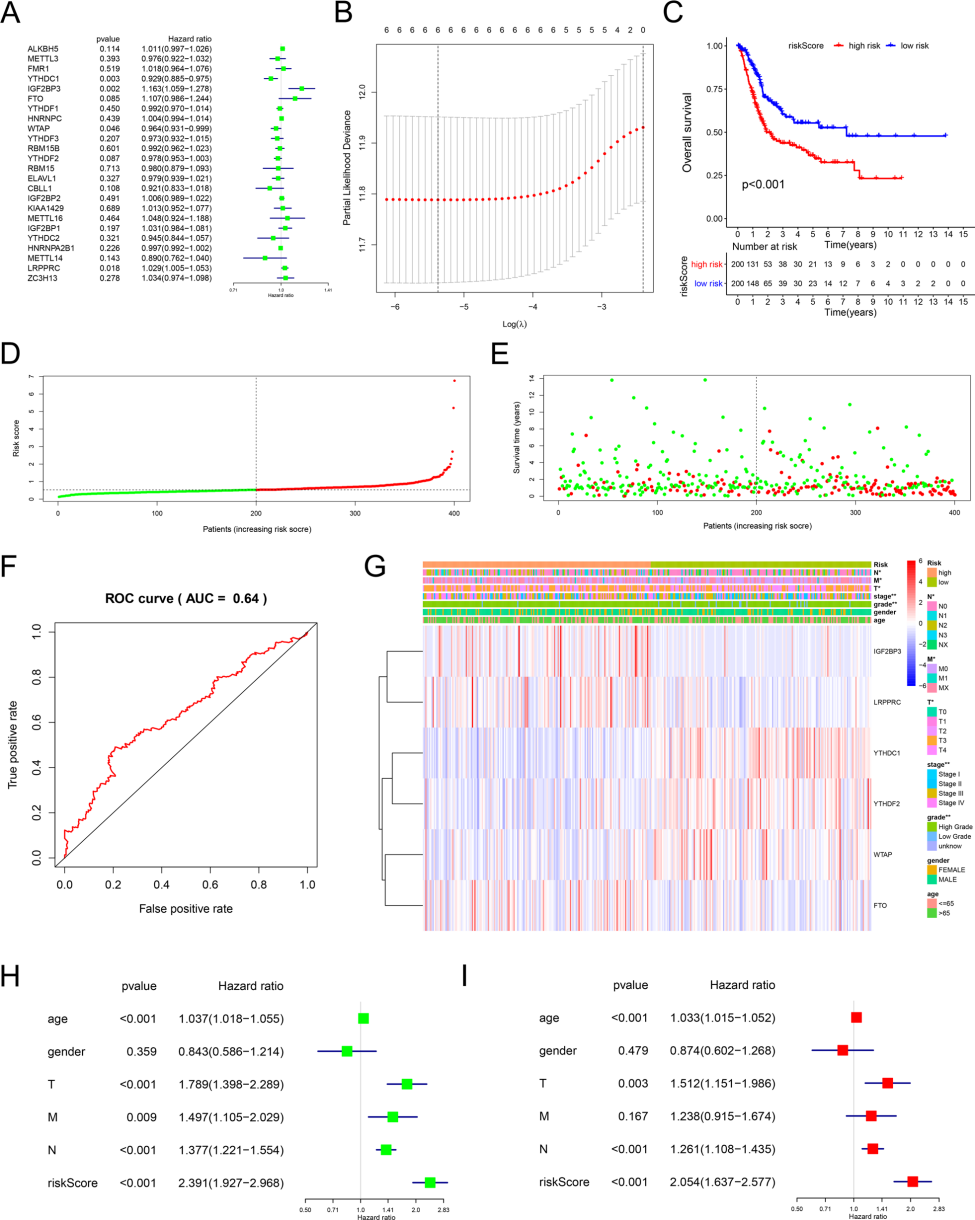
Characteristics of risk score patterns based on m^6^A regulators. **A** The univariable Cox regression analysis for predicting the prognosis of m^6^A regulators. Hazard ratio > 1 represents the risk markers for survival and hazard ratio < 1 represents the protective markers for survival. **B** The most regularized and parsimonious risk score pattern was built by multivariable Cox regression using the least absolute shrinkage and selection operator (LASSO) Cox regression analysis The grey solid vertical lines represent the partial likelihood deviance ± standard error (SE). The black vertical lines are drawn at the optimum values by minimum criteria and 1‐SE criteria. **C** Kaplan–Meier curves for low- and high-risk score patient groups (Log-rank test). **D**, **E** Evaluation of the relationship of the risk score patterns with overall survival status. Red dots represent death and green dots represent alive. **F** Receiver operating characteristic (ROC) curve represents the predictive efficiency of the risk score patterns. **G** Heatmap shows the expression levels of the m^6^A regulators in low- and high-risk score patients with BCa. N status, M status, T status, tumor stage, tumor grade, gender, and age were used as patient annotations. Red represents the high expression of regulators and blue represents the low expression. **H**, **I** Cox regression analyses of the clinicopathological factors and risk score patterns in patients with BCa from TCGA. Univariable Cox regression analyses (**H**), Multivariable Cox regression analyses (**I**).*P < 0.05, **P < 0.01

To further explore the prognostic value of m^6^A regulators in BCa, we first performed a univariable Cox regression analysis on the expression levels of m^6^A regulators. The results showed that high expression of *IGF2BP3* (hazard ratio [HR] = 1.2, 95% confidence interval [CI] = 1.1–1.3) and *LRPPRC* (HR = 1.0, 95% CI = 1.0–1.1) had worse survival outcomes in patients with BCa, whereas *YTHDC1* (HR = 0.9, 95% CI = 0.9–1.0) and *WTAP* (HR = 1.0, 95% CI = 0.9–1.0) were regarded as protective markers for BCa (Fig. 4A). However, high expression of ZC3H13 seemed to have worse survival outcomes in patients with BCa regardless of P value.

LASSO Cox regression analysis was performed to determine the optimal genes for selecting predictors and building the most regularized and parsimonious risk score pattern, and we only chose the prognostic value of m^6^A regulators P < 0.1 for further analysis. The genes and their coefficients obtained from the LASSO analysis were used to calculate the risk scores for individual patients (Fig. 4B, Additional file 1: Fig. S5A, Table S3). The final LASSO model with the best optimal lambda included six m^6^A regulators (*IGF2BP3*, *LRPPRC*, *YTHDC1*, *YTHDF2*, and *WTAP*). To investigate the prognostic value of the risk score pattern, BCa patients were divided into low-and high-risk groups, and the Kaplan–Meier curve revealed that the patients in the high-risk group had a worse survival than patients in the low-risk group (Fig. 4C). As shown in Fig. 4D, E, green (low risk or alive) and red (high risk or dead) dots demonstrated significant differences between the low-and high-risk groups. ROC analysis showed a risk score pattern with AUC = 0.64, indicating that the risk score could predict the OS of patients with BCa (Fig. 4F). Next, we explored the correlation between the risk score pattern and clinical characteristics, and the risk score pattern was related to tumor grade, tumor stage, T status, M status, and N status (Fig. 4G and Additional file 1: Table S4). Moreover, the expression of risk genes (*IGF2BP3*, *LRPPRC*, and *FTO*) was higher in high-rsik patients, while *YTHDC1, YTHDF2*, and *WTAP* tended to be expressed in the low-risk group. Univariable and multivariable Cox analyses were performed to determine the independent prognostic value of the risk score pattern. Patient age, tumor T status, N status, and risk score were independent prognostic predictors in patients with BCa (Fig. 4H).

To better understand the function of the risk score pattern, we analyzed the GO analysis of DEGs based on expressions in low-and high-risk score groups. GO analysis indicated that upregulated genes in the high-risk group were enriched in malignancy-related biological processes, including extracellular matrix organization, extracellular structure organization, and antimicrobial humoral response, and downregulated genes were enriched in hormone metabolic processes and terpenoid metabolic processes (Additional file 1: Fig. S5B, C). GSVA enrichment analysis was conducted to explore the different pathways between the two groups. The results revealed that the high-risk group was significantly related to the malignant pathways, including gap junction, focal adhesion, and ECM receptor interaction (Additional file 1: Fig. S5D, E). Then, we investigated the distribution differences of somatic mutation between low and high risk score by using “maftools” R package. As shown in Additional file 1: Fig. S5F–K, the high-risk score group exhibited more somatic mutation burden than the low-risk score group, and missense mutation was the most common variant classification; the most common variant type was SNP, and C>T transversion was the most common type of SNV class. Moreover, the top three mutated genes were *TP53*, *TTN*, and *KDM6A* in the low-risk group and *TP53*, *TTN*, and *KMT2D* in the high-risk group, respectively. Taken together, the risk score pattern based on m^6^A regulators could be regarded as an independent prognostic factor in patients with BCa, and the high-risk group gained more malignant behaviors and more mutation burden.

### Characteristics of immune landscape with risk score pattern

**Fig. 5:**
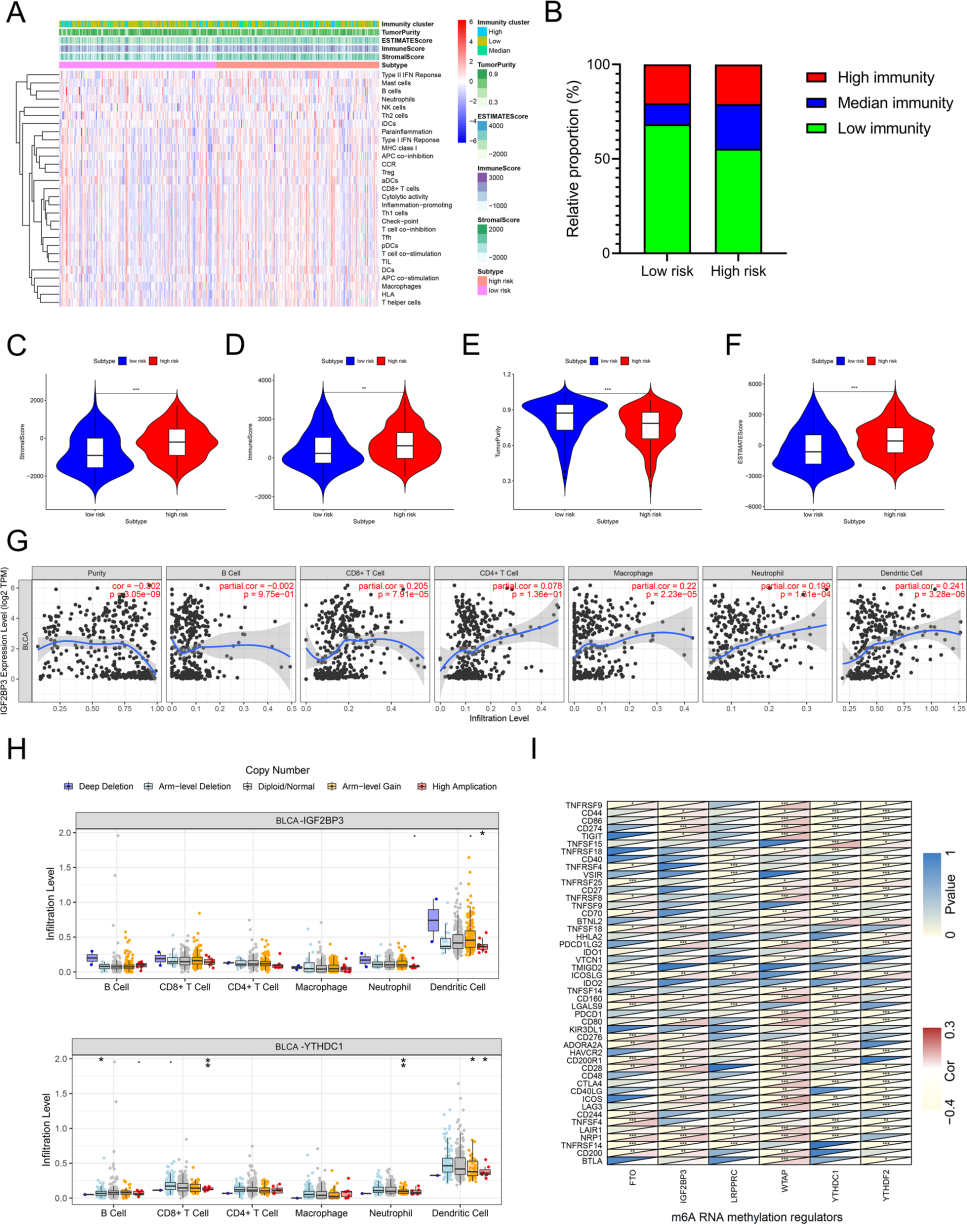
Characteristics of immune signatures with m^6^A regulators and risk score patterns. **A** Heatmap shows the enrichment of 29 immune signatures in two risk score groups. Immunity cluster, tumor purity, ESTIMATE score, immune score, and stromal score were used as patient annotations. **B** The proportion of patients from different immunity clusters in low and high risk groups. **C**–**F** Violin plot shows the different status of scores in risk score groups. Stromal score (**C**), immune score (**D**), tumor purity (**E**), and ESTIMATE score (**F**). **G** The association of insulin-like growth factor 2 mRNA-binding protein 3 (IGF2BP3) expression levels with 6 immune cells and the tumor purity. The data was obtained from the Tumor Immune Estimation Resource (TIMER) website (https://cistrome.shinyapps.io/timer/). **H** The association of m^6^A regulators mutations with 6 immune cells. The data was obtained from the TIMER website (https://cistrome.shinyapps.io/timer/). **I** The association between m^6^A regulators in risk score patterns with immune checkpoints. Red at the bottom right corner represents the positive correlation, and yellow represents the negative correlation. Yellow also represents statically difference at the top left corner. *P < 0.05, **P < 0.01, ***P < 0.001

To explore the potential relationship between immunity and risk score pattern, we first divided samples into three clusters, immunity low, median and high using the ssGSEA score to quantify the immune cell types, functions and pathways, and the differences in 29 immune-associated gene sets were shown in three distinct immunity clusters (Additional file 1: Fig. S6A, B). Next, we investigated the correlation between the immune landscape and the risk score pattern. As shown in Fig. 5A, the enrichment of the immune landscape in the high-risk group was higher than that in the low-risk group. Moreover, the percentage of low immunity samples in the high-risk group was significantly lower than that in the low-risk group and more median immunity samples in the high-risk group than that in the low-risk group (Fig. 5B). In addition, comparing the stromal score, immune score, tumor purity, and ESTIMATE score between the two distinct risk score groups, we found that the high-risk group had significantly higher stromal scores, immune scores, and ESTIMATE scores, and lower tumor purity (Fig. 5C–F). Taken together, these results suggest that the risk score pattern has a strong relationship with the immune landscape, and the potential mechanisms of m^6^A regulators in tumorigenesis and progression may be associated with tumor immunity.

Next, we explored the immune characteristics of independent m^6^A regulators in the risk group using the TIMER database to investigate the correlation between m^6^A regulator expression and immune cells, including B cells, CD8 + T cells, CD4 + T cells, macrophages, neutrophils, dendritic cells, and tumor purity. As shown in Fig. 5G and Additional file 1: Fig. S6C, the expression of *IGF2BP3* was positively correlated with macrophages, neutrophils, and dendritic cells and negatively correlated with tumor purity. The expression of *LRPPRC* was positively correlated with B cells, CD8 + T cells, neutrophils, and dendritic cells, and negatively correlated with CD4 + T cells. As for *FTO*, B cells, CD8 + T cells, macrophages, neutrophils, and dendritic cells were identified as significant co-expression cells. The expression of *WTAP* was negatively correlated with tumor purity, but positively correlated with CD8 + T cells, CD4 + T cells, neutrophils, and dendritic cells. The expression of *YTHDC1* was only correlated with tumor purity, B cells, and macrophages. As for *YTHDF2*, tumor purity, B cells, CD8 + T cells, and neutrophils showed a strong correlation. The SCNA module, which was defined by GISTIC 2.0, was conducted to provide a comparison of immune infiltration levels in BCa with different somatic copy number alterations for m^6^A regulators. As shown in Fig. 5H and Additional file 1: Figure S6D, *IGF2BP3* amplification was associated with dendritic cells.*YTHDC1* deletion was related to B cells, while amplification was related to CD8 + T cells, neutrophils, and dendritic cells. Moreover, *FTO* amplification had a connection with B cells and macrophages, and deletion had a connection with CD8 + T cells. Interestingly, *LRPPRC* deletion and amplification were both associated with CD4 + T cells, neutrophils, and dendritic cells. The *YTHDF2* mutation was associated with immune cells, except for CD8 + T cells and macrophages. The *WTAP* mutation is only related to CD4 + T cells and neutrophils. Furthermore, we investigated the co-expression of m^6^A regulators in the risk score model and several immune checkpoints (Fig. 5I). The results indicate that m^6^A regulators are correlated with most immune checkpoints, including *PD-L1* (also known as *CD274*). In summary, these results strongly indicate that the risk score pattern based on m^6^A regulators is significantly correlated with the tumor immune landscape.

### Construction and validation of nomogram

**Fig. 6:**
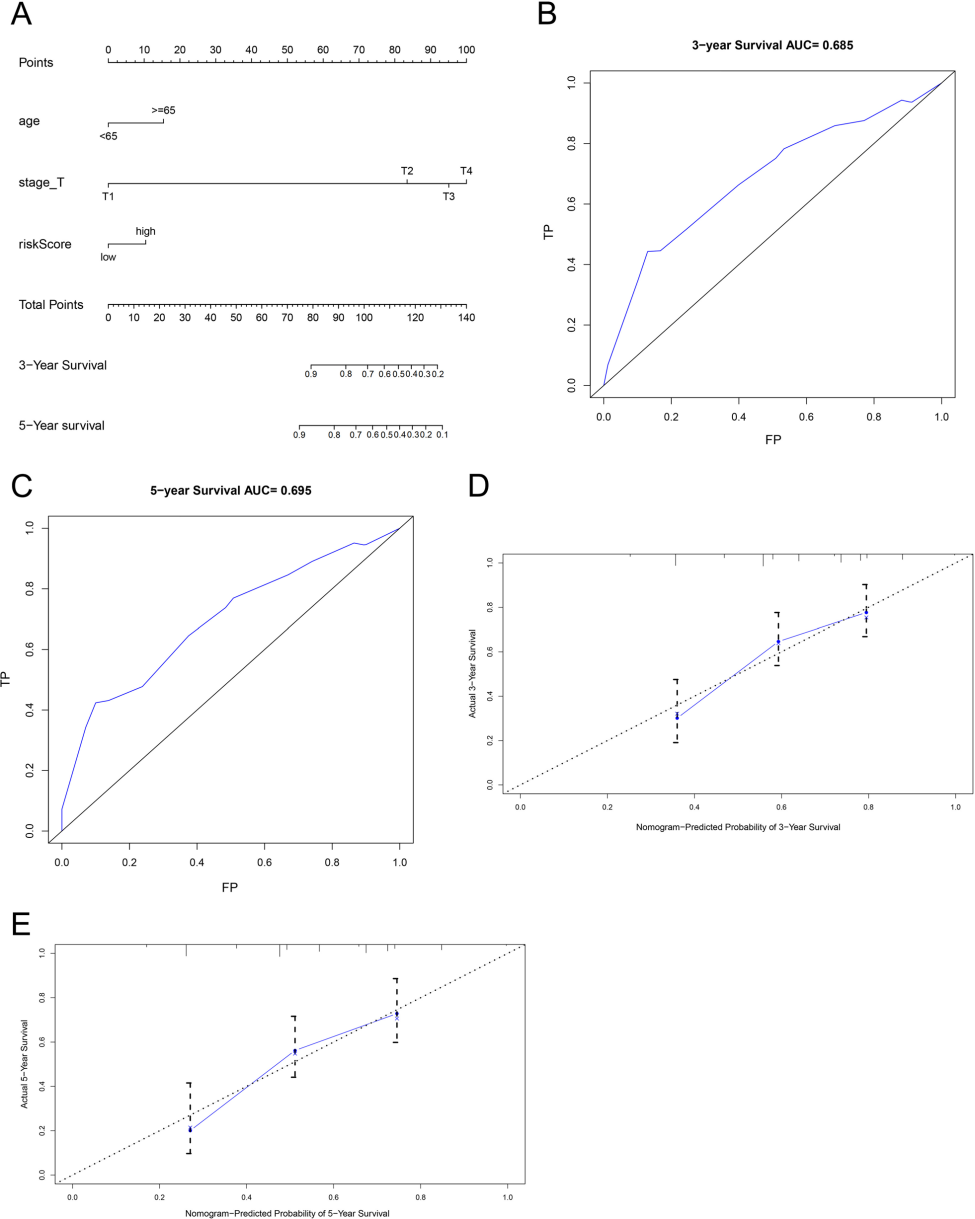
Construction of a nomogram to predict the prognosis of individual patients. **A** Baseline nomogram for predicting the probability of patients with 3- and 5-years was constructed from 3 clinicopathological parameters. **B**, **C** ROC curves of the nomogram for predicting (**B**) 3- and (**C**) 5-year overall survival (OS) status. **D**, **E** The calibration plots for predicting OS of patients at 3- (**D**) and 5-years (**E**), nomogram‐predicted survival probability is plotted on the x‐axis; actual survival probability is plotted on the y‐axis. The solid line represents our nomogram and the vertical bars represent 95% confidence intervals

A nomogram was established based on the independent factors using a multivariable Cox regression model to predict OS in patients with BCa (Fig. 6A). The AUCs of the nomogram for predicting the 3- and 5-year OS were 0.69 and 0.70, respectively (Fig. 6B, C). The c-index of the nomograms for OS in the training set was 0.68. As shown in Fig. 6D, E, calibration plots were generated to validate the similarities between the actual survival rate and the survival prediction by the nomogram, and the results demonstrated that the 3- and 5-year survival rates predicted by the nomogram were closely corresponded with the actual survival rates in the training set.

Moreover, 30 percent of patients with BCa were selected in the internal validation set. The AUCs in the validation set for predicting the 3- and 5-year OS were 0.75 and 0.72, respectively (Additional file 1: Fig. S7A, B). The c-index of the nomogram in the validation set was 0.688. The results of the calibration plot suggested that the predicted 3- and 5-year survival rates were consistent with the actual survival rate within an acceptable margin of error in patients with BCa (Additional file 1: Fig. S7C, D).

### Characteristics of *IGF2BP3* expression in cancers

**Fig. 7:**
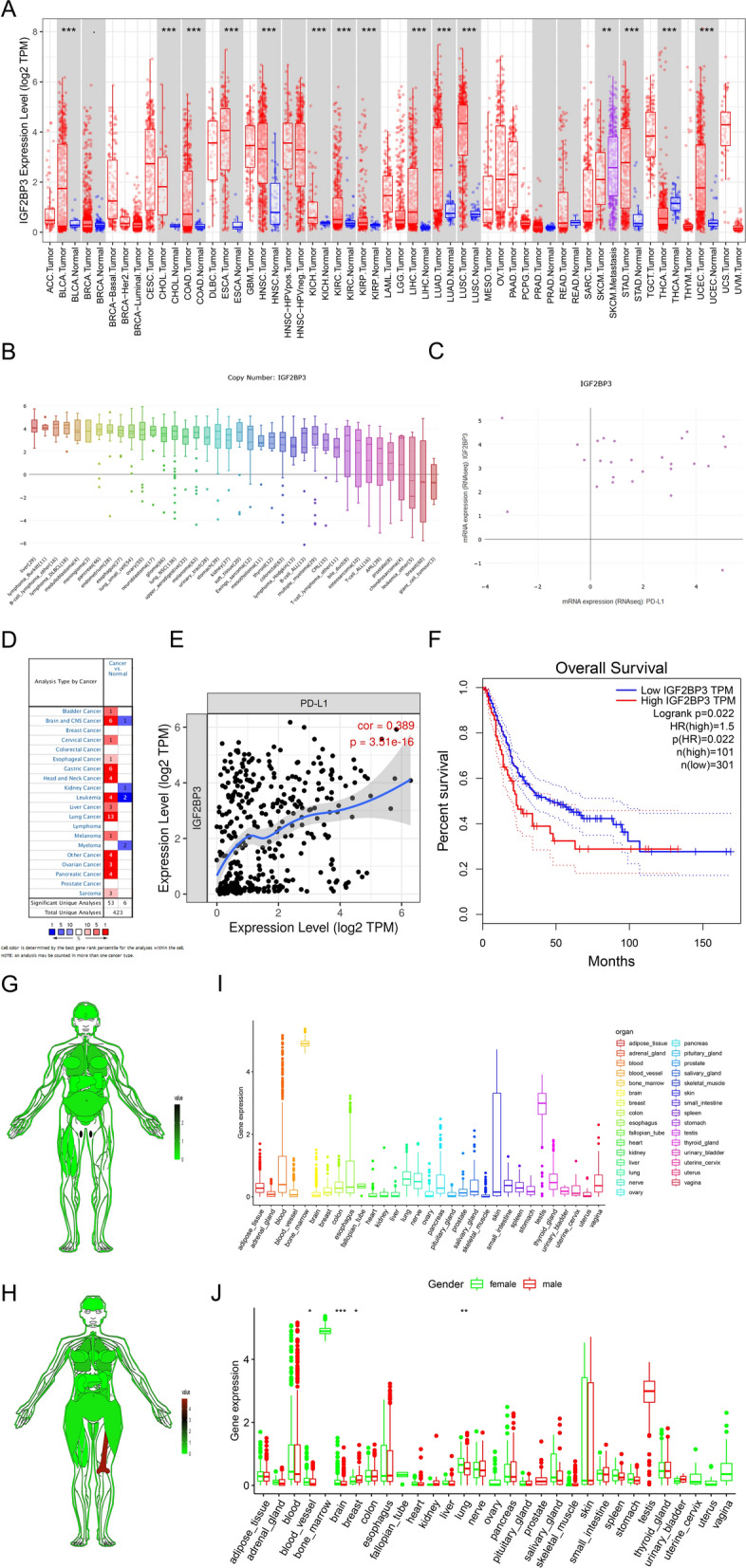
The expression levels and prognostic value of IGF2BP3. **A** The comparison of IGF2BP3 expression levels between the tumor and normal tissues. **B** IGF2BP3 expression levels in different tumor cell lines from the Cancer Cell Line Encyclopedia (CCLE) database. **C** The correlation between the programmed cell death ligand 1 (PD-L1) and IGF2BP3 mRNA expression levels from the CCLE database. **D** IGF2BP3 expression levels in the Oncomine database. P value threshold was set 0.05 and the fold-change threshold was set 2.0. The number in the colored cells represents the number of studies meeting the thresholds. The printed red (over-expression) or blue colors (under-expression) indicate a significant association. **E** The relation of IGF2BP3 with PD-L1 from the TIMER website. **F** The Kaplan–Meier curve shows the prognostic values of IGF2BP3 in patients with BCa obtained via Gene Expression Profiling Interactive Analysis (GEPIA), which was based on TCGA database. **G**, **H** The anatomical structure showed the expression levels of the IGF2BP3 in the normal organ tissues in males (**G**) and females (**H**). **I** Histogram visualizing the expression levels of IGF2BP3 in normal organ tissues. **J** Histogram visualizing the expression levels of IGF2BP3 in normal organ tissues between the females and males. *P < 0.05, ***P < 0.001

Because of the important role of *IGF2BP3* in risk score patterns, we used TCGA, GTEx CCLE, and Oncomine datasets to further understand *IGF2BP3* in normal and tumor tissues. As shown in Fig. 7A, the expression of *IGF2BP3* was higher in BCa, cholangiocarcinoma (CHOL), colon adenocarcinoma (COAD), esophageal carcinoma (ESCA), head and neck squamous cell carcinoma (HNSC), kidney chromophobe (KICH), kidney renal clear cell carcinoma (KIRC), kidney renal papillary cell carcinoma (KIRP), liver hepatocellular carcinoma (LIHC), lung adenocarcinoma (LUAD), lung squamous cell carcinoma(LUSC), stomach adenocarcinoma (STAD), uterine corpus endometrial carcinoma (UCEC), and low expression in thyroid carcinoma (THCA), compared to their corresponding normal tissues (Fig. 7A). Moreover, the CCLE dataset was used to evaluate the expression levels of *IGF2BP3* in various tumor cell lines. The results showed that the top three expression levels in tumor cell lines were liver cancer, lymphoma, and medulloblastoma. *IGF2BP3* seemed to be positively associated with *PD-L1* expression in BCa cell lines (Fig. 7B, C).

The Oncomine database was used to determine the expression level of *IGF2BP3*. And as shown in Fig. 7D, *IGF2BP3* in most cancer types showed high expression levels, except for kidney cancer and myeloma, which were opposite to the TCGA database. Furthermore, the correlation between *IGF2BP3* and *PD-L1* in patients with BCa showed a trend similar to that of bladder cancer cell lines from CCLE (Fig. 7E). Next, we investigated the prognostic value of *IGF2BP3* in BCa using the GEPIA website, and the results revealed that patients with high expression of *IGF2BP3* had worse prognosis in BCa (Fig. 7F). The GETx database indicated that the expression level of *IGF2BP3* in male and female bone marrow was significantly high. To compare the expression differences between males and females, and there was no difference in the expression of *IGF2BP3* in most female and male tissues, except for blood vessels, brain, breast, and lung (Fig. 7G–J). Taken together, these results reveal that the expression of *IGF2BP3* is high in various tumors and is associated with *PD-L1*, which may be a potential target for anti-*PD-L1* immunotherapy.

### The correlation between* IGF2BP3* and *PD-L1* in BCa cells and tumor specimen

**Fig. 8:**
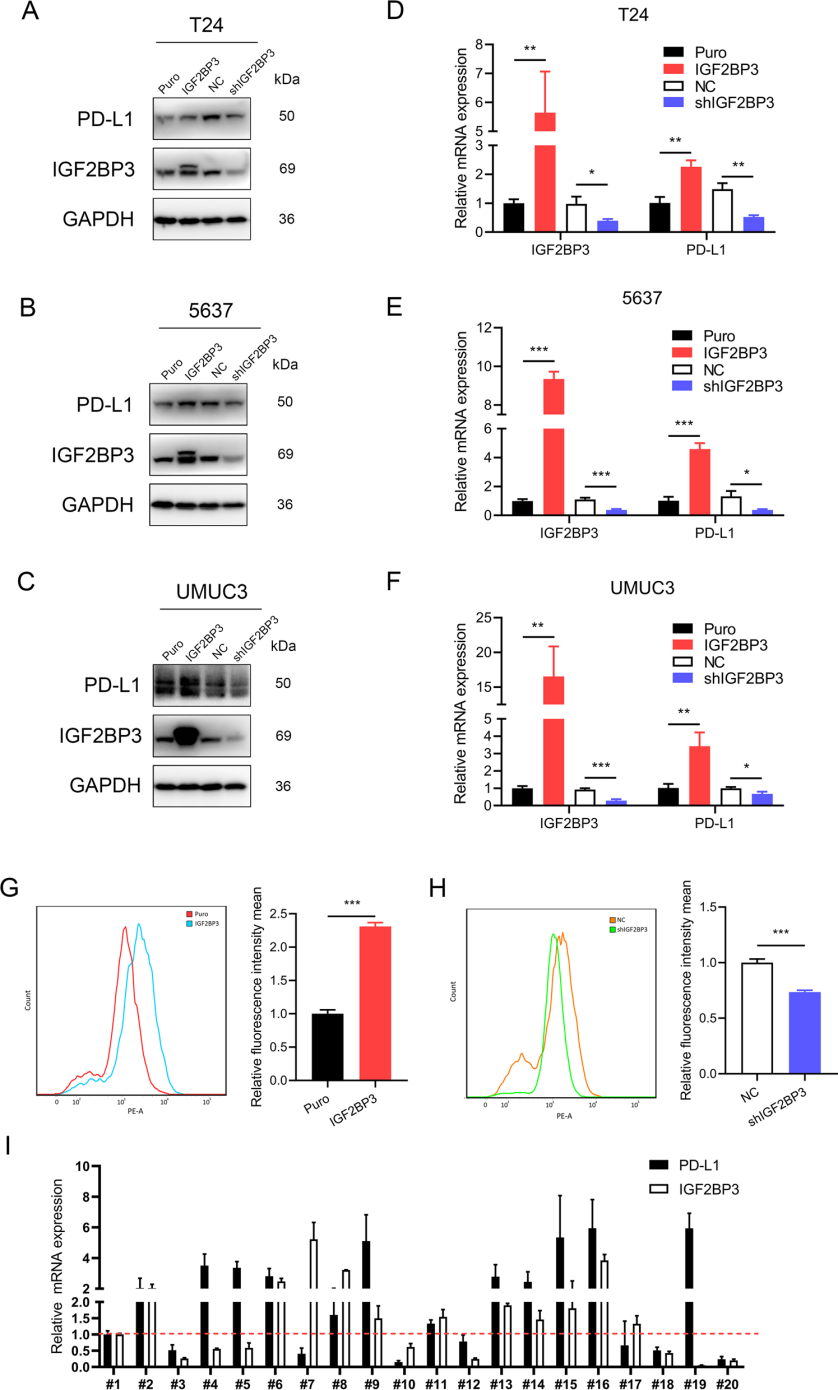
The association between IGF2BP3 and PD-L1. **A**–**C** The protein expression levels of IGF2BP3 and PD-L1 in BCa cells were determined by western blotting. T24 (**A**), 5637 (**B**) and UMUC3 (**C**). **D**–**F** The mRNA levels of IGF2BP3 and PD-L1 in indicated cells were detected by quantitative polymerase chain reaction (qPCR), Puro was set as 1, Puro vs IGF2BP3, negative control (NC) vs shIGF2BP3. T24 (**D**), 5637 (**E**) and UMUC3 (**F**). **G**–**H** Two representative flow cytometry staining of PD-L1 in indicated T24 cells are shown (left), and quantification of PD-L1 fluorescence intensity is shown (right), Puro and NC were set as 1, Puro vs IGF2BP3, NC vs shIGF2BP3. **I** The mRNA levels of IGF2BP3 and PD-L1 in tumor specimens were detected by qPCR, patient #1 was set as 1. All quantification analyses were based on independent triplicate experiments. Error bars represent the standard deviation (SD). *P < 0.05, **P < 0.01, ***P < 0.001, based on Student’s t test

Given that *IGF2BP3* had a strong correlation with *PD-L1* analyzed using a public database, we examined the expression levels of *IGF2BP3* and *PD-L1* in vitro. Stable *IGF2BP3* overexpression and knockdown of T24, 5637, and UMUC3 cells were established, and the results revealed that overexpression of *IGF2BP3* significantly increased, while knockdown of *IGF2BP3* decreased both the protein and mRNA levels of *PD-L1* in BCa cells (Fig. 8A–F). Further, flow cytometric assay showed that overexpression of *IGF2BP3* significantly enhanced membrane-bound *PD-L1* expression, and knockdown of *IGF2BP3* decreased membrane-bound *PD-L1* expression in T24 cells (Fig. 8G–H). The correlation between *IGF2BP3* and *PD-L1* expression was analyzed using BCa specimens. As shown in Fig. 8I, the positively correlated expression between *IGF2BP3* and *PD-L1* was found in 14/20 (70%) tumor specimens. Taken together, these data demonstrate that *IGF2BP3* regulates both total and membrane-bound *PD-L1* expression levels in BCa.

## Discussion

Although several studies have explored the m^6^A regulators in tumorigenesis and tumor development, the comprehensive analysis of m^6^A regulators with tumor immune landscape in bladder cancer has been poorly investigated. Here, we reveal that distinct clusters and risk groups are associated with tumor immunity and have prognostic value for patients with BCa. Furthermore, *PD-L1* is identified as a potential target of *IGF2BP3*, and *IGF2BP3* can regulate both total and membrane-bound *PD-L1* expression levels.

Modified RNA bases have been discovered for over six decades. After the first RNA demethylase (*FTO*) was identified, the research field was revived, and the formation of m^6^A is a reversible process [6, 36]. The m^6^A regulators can be divided into three functional groups: writers, erasers, and readers. Writing is the process of adding methylated modifications to RNA, including *METTL3*, *METTL14*, *METTL16*, *KIAA1429*, *WTAP*, *RBM15*, *RBM15B*, *CBLL1*, and *ZC3H13* [5, 22, 24], while the reversible process is mediated by erasers, including the *FTO* and *ALKBH5*. Moreover, m^6^A indirectly affects RNA processing by recruiting specific reader proteins, including nuclear m^6^A readers, *YTHDC-1*, *HNRNPA2B1*, *HNRNPC*, etc., and cytoplasmic m^6^A readers, *YTHDF-1/2/3* and *YTHDC2*.Increasing evidence has shown that m^6^A regulators play a crucial role in various pathophysiological processes, including circadian rhythms, spermatogenesis, DNA damage response, tumorigenesis, and tumor progression [37–40].

The TME consists of immune cells and non-immune cells, which could play a crucial role in tumor growth and progression, and tumor-infiltrated immune cells are highly associated with tumorigenesis, angiogenesis, and metastasis [41]. Meanwhile, the imbalance between tumor cell growth and elimination might activate immunosurveillance. As the understanding of complexity of TME has deepened, more evidence has shown that tumor-infiltrating cells play either tumor-suppressive or tumor-promoting roles, thus influencing cancer initiation and progression. For instance, M1 macrophages mainly produce pro-inflammatory cytokines that potentiate the anti-tumor immune response, while M2 macrophages promote ECM deposition and immunosuppression [42]. Therefore, understanding the crosstalk between TME and tumor cells might be useful for assessing the prognosis and improving the response rate of immunotherapy for individual patients with various cancers.

Bladder cancer is highly correlated with immunotherapy, including *anti-PD-L1* and *anti-CTLA4* becomes a hotspot in advanced BCa treatment. The Food and Drug Agency (FDA) and the European Medicines Agency (EMA) granted accelerated approval to atezolizumab and pembrolizumab as first-line metastatic cisplatin-unfit BCa. However, *anti-PD-L1* treatment showed limited efficacy in the first-line phase III clinical trials [43], with a relatively low response rate of approximately 20% [44]. Moreover, several studies have recently demonstrated that *PD-1* and *PD-L1* expression are not reliable biomarkers for predicting the benefits of immunotherapy [45, 46]. A retrospective study demonstrated that patients progressing to frontline *PD-1/PD-L1* immunotherapy were even at risk of early death, excluding them from experiencing potential benefit from subsequent systemic treatment [47].

Several studies have demonstrated the function of m^6^A regulators in bladder cancer. For instance, methyltransferase-like (*METTL)-3* might act as an oncogene by interacting with the DiGeorge syndrome critical region 8 (*DGCR8*) and accelerating the pri-miR221/222 maturation to promote tumor proliferation [48]. Meanwhile, *METTL3* plays a role in BCa progression by promoting the cancer cell growth and invasion by regulating a network that involves the *AF4/FMR2* family member 4 (*AFF4*), nuclear factor-kappa B (*NF-κB*), and *Myc* [49]. Another mechanism of the m^6^A regulator *METTL3/* YTH N^6^-methyladenosine RNA binding protein (*YTHDF2*) mA axis directly degrades the mRNAs of SET domain containing 7 (*SETD7*) and Kruppel-like factor 4 (*KLF4*), contributing to the progression of BCa [50]. Analysis of the expression levels of *METTL3* and *CDCP1* in patients with BCa revealed that *METTL3* and *CDCP1* were strongly upregulated in the tumor samples, and the *METTL3-CDCP1* axis could increase the tumor proliferation, migration, and invasion [51].

Most studies have focused on m^6^A regulators or immunotherapy. However, the correlation between m^6^A regulators and tumor immunity has not been fully recognized, and only a few studies have demonstrated the potential relationship between m^6^A regulators and TME anti-tumor immune responses in various cell types, such as gastric cancer, melanoma, and dendritic cells [22, 52, 53]. Here, we first identified two distinct m^6^A clusters and constructed a risk score pattern based on m^6^A regulators to reveal the potential pathways and functional processes, predict the prognosis of patients with BCa, and investigate the correlation between m^6^A regulators and tumor immunity. Moreover, we analyzed one of the m^6^A regulators in pattern, *IGF2BP3*, and identified its expression level, prognostic value, and association with *PD-L1*. Clarifying the role of risk score pattern with TME will contribute to broadening the understanding of TME antitumor immune response and suggest appropriate effective immunotherapy strategies for individual BCa patients.

Although the prognostic value of the m^6^A cluster was limited, the m^6^A cluster was associated with tumor grade, and two clusters showed significantly distinct pathway enrichment. Cluster 1 was characterized by metabolism, and cluster 2 was characterized by DNA damage. And the clusters based on m^6^A regulator expression could provide a few fresh outlooks for further study. Moreover, the correlation analysis revealed that the most significant positive and negative relevance were *METTL3* with *YTHDF3* and *IGF2BP2*, respectively, showing different functions in BCa. The function of m^6^A regulators primarily depends on reader proteins. *YTHDF2* could induce mRNA degradation and *YTHDF1* and *YTHDF3* could initiate mRNA translation, while the *IGF2BP* family could enhance the stability of target mRNA [54–57]. A comprehensive analysis of m^6^A regulators revealed that the mRNA expression of *METTL4* and *YTHDF3* was higher in high-grade tumors than in low-grade tumors, and *YTHDC1* was upregulated in the I/II stage, compared to the III/IV stage [58].

Furthermore, the risk score pattern based on 6 m^6^A regulators revealed its prognostic value for OS in patients with BCa, and the risk score pattern was highly associated with pathological features, such as T status, M status, N status, and tumor grade. Moreover, in the present study, we found an association between risk score pattern and TME. High immune score, high stromal score, high ESTIMATE score, and low tumor purity were found in the high-risk score group. We next found that the expression and mutation of individual m^6^A regulators in the risk score pattern was associated with immune cells and immune checkpoints, which could underlie part of the mechanism of the risk score pattern. A nomogram was constructed to evaluate the prognostic value of individual patients for predicting 3- and 5-year survival times. If the physicians were able to estimate whether individual patients had shorter or longer than the median OS according to their expression of m^6^A regulators in tumor tissues, it would be useful for patients with different treatment strategies [59].

*IGF2BP3* was highly expressed in the high-risk group, and a recent study demonstrated that *IGF2BP3* could be regarded as an independent prognostic factor in NMIBC, which could present a subgroup of patients with high probability of relapse, progression, and metastasis [60]. A comprehensive study has reported that the expression of *IGF2BP3* was detected in 76 different normal tissue types and 3889 cancer samples from 95 different tumor categories, *IGF2BP3* overexpression has been found in various cancer types, and *IGF2BP3* is typically associated with aggressive tumor features [61]. *IGF2BP3* has been shown to directly interact with *ULBP2* mRNA, thereby reducing *ULBP2* surface expression. *IGF2BP3* indirectly interacts with MICB. The *IGF2BP3*-mediated pathway leads to impaired NK cell recognition of transformed cells to facilitate tumor immune escape [62]. In the present study, we found a positive correlation between *IGF2BP3* and *PD-L1*, and *IGF2BP3* could regulate total and membrane-bound *PD-L1* expression levels in BCa cells, which implies the potential role of *IGF2BP3* in *anti-PD-L1* immunotherapy.

This study has several limitations. First, because of the limited clinical database on BCa, only TCGA patients with clinical characteristics were included. Second, immunohistochemical staining of m^6^A regulators was obtained from the public database, and the protein levels of m^6^A regulators will be explored in further studies. Third, our nomogram only underwent internal validation; it could be more powerful to obtain an external validation with a large multicenter cohort. Despite considering the limitations of the present study, our findings provide novel insights for m^6^A regulator clusters, risk score based on m^6^A regulators, and identified the association between tumor immunity and m^6^A regulators.

In summary, the present study investigated the cluster and prognosis of m^6^A regulators in BCa and found that the expression of m^6^A regulators is highly correlated with clinicopathological characteristics. We also constructed a risk score pattern and nomogram to evaluate the OS of patients with BCa. Moreover, we illustrated the relationship between m^6^A regulators and the TME. Therefore, our study provides important ideas for improving the clinical outcomes of patients with BCa, which may be used to develop different immunotherapies based on the expression levels of m^6^A regulators.

## Supplementary Information

**Additional file 1.** Supplementary figures and tables.

## Data Availability

The data and materials in the current study are available from the corresponding author on reasonable request.
