# Supplementary material for: Comprehensive analysis of N6-methyladenosine regulators with the tumor immune landscape and correlation between the insulin-like growth factor 2 mRNA-binding protein 3 and programmed death ligand 1 in bladder cancer
Source: Cancer Cell Int. 2022 Feb 11;22:72. doi: 10.1186/s12935-022-02456-7 (PMC8840771; doi:10.1186/s12935-022-02456-7)
Supplement: Supplementary file 1 — Additional file 1. Supplementary figures and tables. [file 12935_2022_2456_MOESM1_ESM.docx]

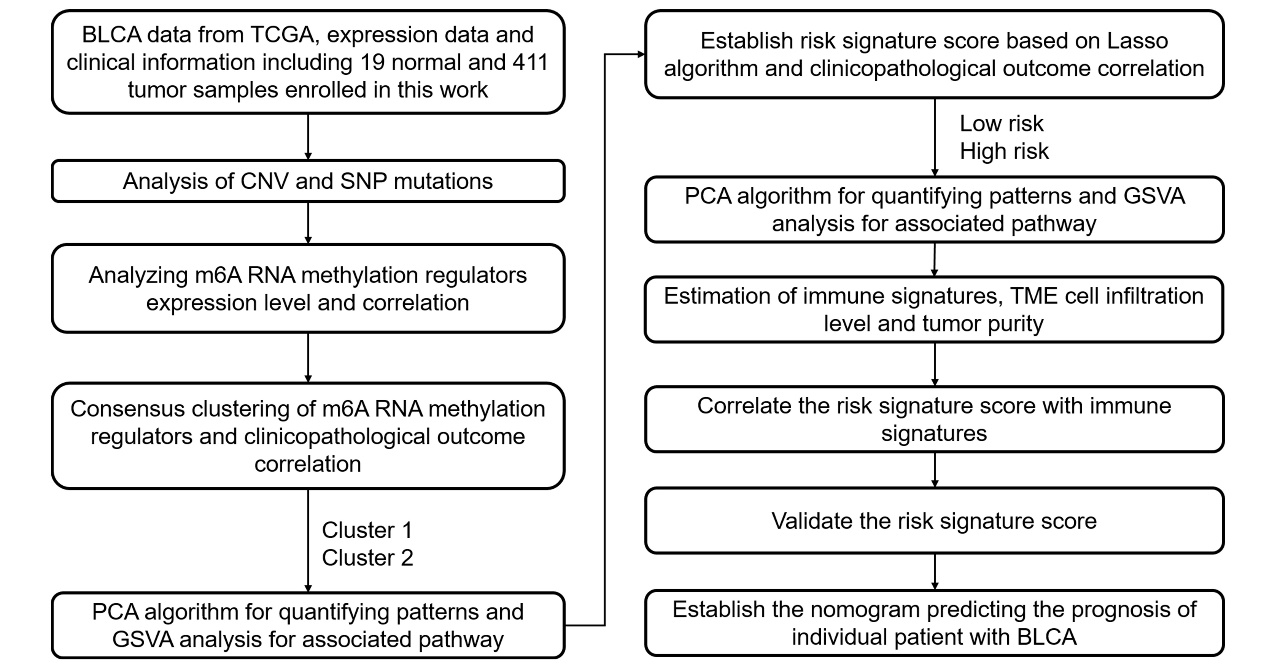


Figure S1 The work flow of present study.


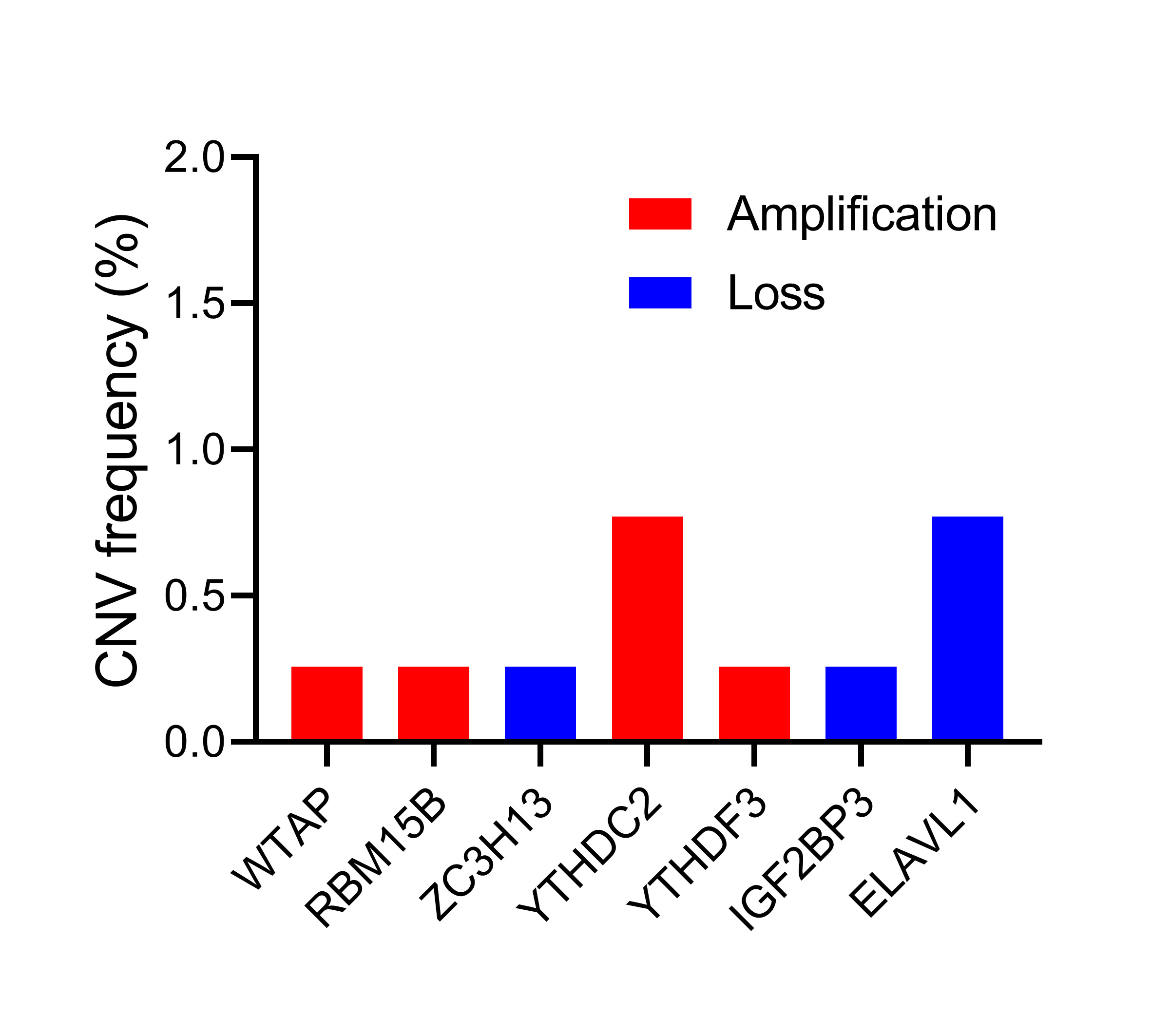


Figure. S2 The CNV variation frequency of m6A regulators in normal bladder tissues from TCGA. The blue column represents deletion frequency, and the red column represents amplification frequency.


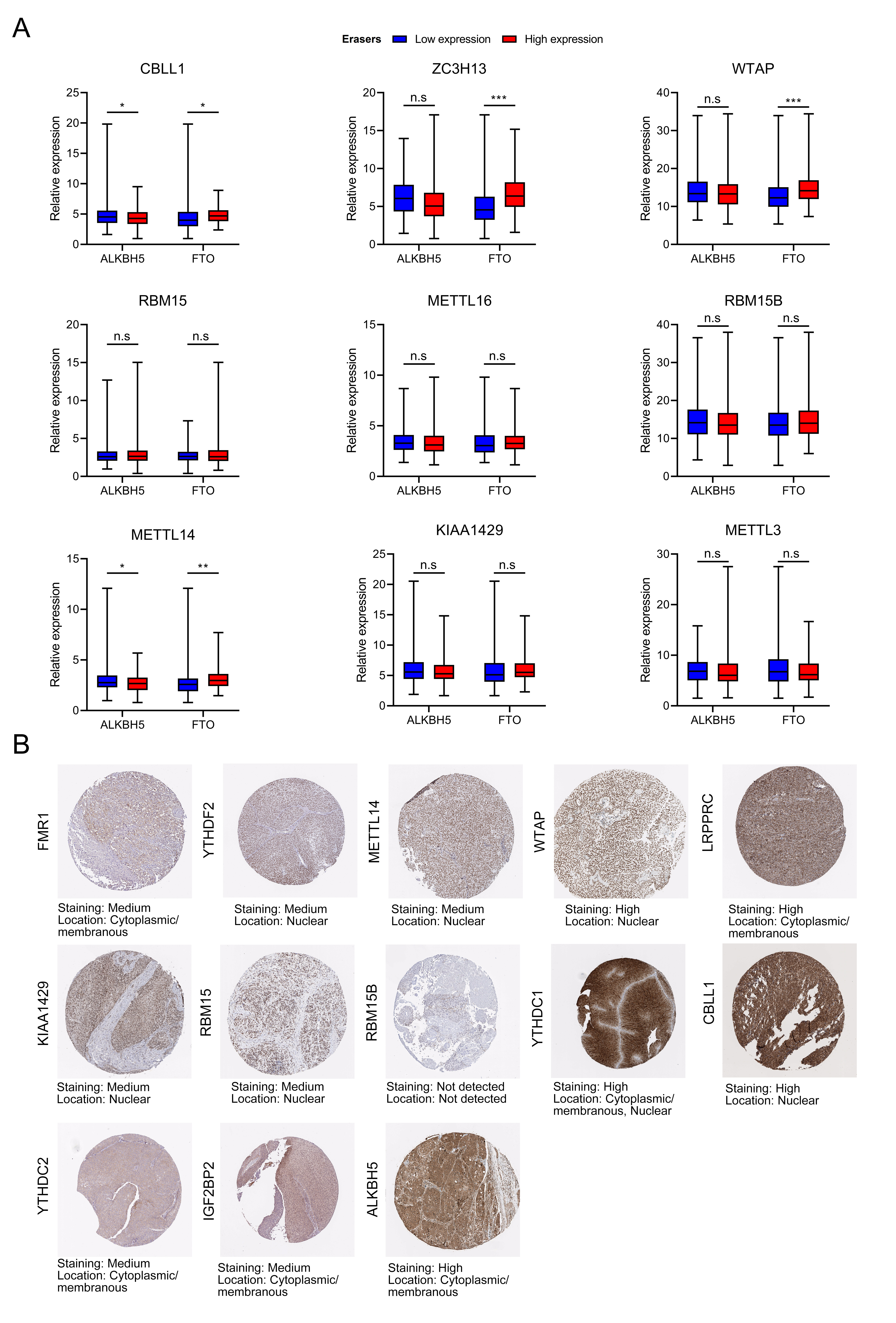


Figure. S3 Profiles of expression level of 24 m6A methylation regulators in bladder cancer. (A) Difference in the writer gene expression between low and high eraser gene expression groups. Writer gene, CBLL1, ZC3H13, WTAP, RBM15, METTL16, RBM15B, METTL14, KIAA1429 and METTL3; Eraser gene, ALKBH5 and FTO. The upper and lower ends of the boxes represented range of values. The lines in the boxes represented median value *P < 0.05; **P < 0.01; ***P < 0.001, n.s=not significant. (B) The protein level of m6A regulators detected by immunohistochemistry staining of were from The Human Protein Atlas.


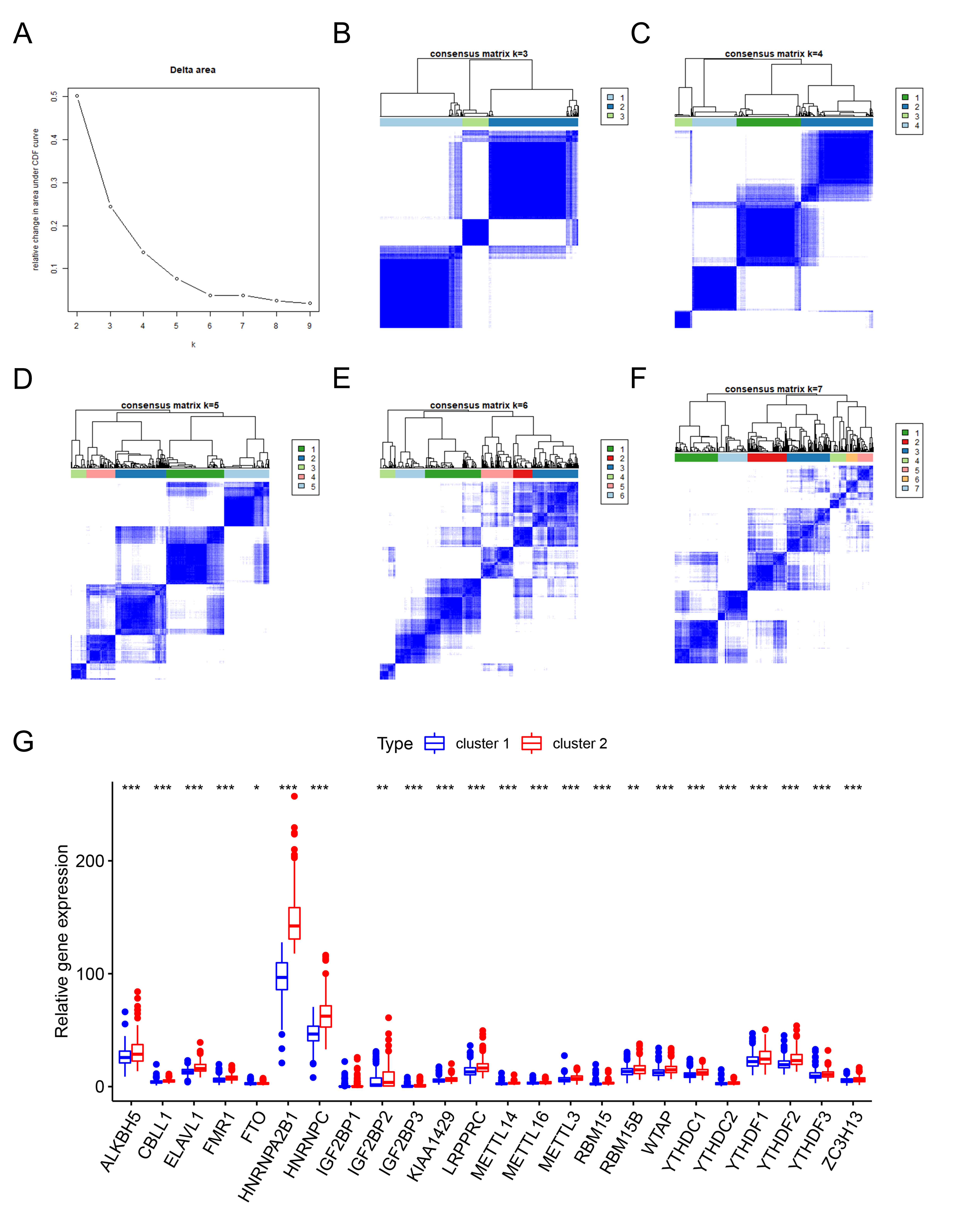


Figure. S4 Identification consensus clusters by m6A regulators. (A) Relative change in delta area under CDF curve for k=2–9 (B-F) Consensus clustering matrix for k=3-7. (G) Box plots of m6A RNA expression between two clusters. m6A regulators in cluster 1 were marked with blue, and those in cluster 2 were marked with red.


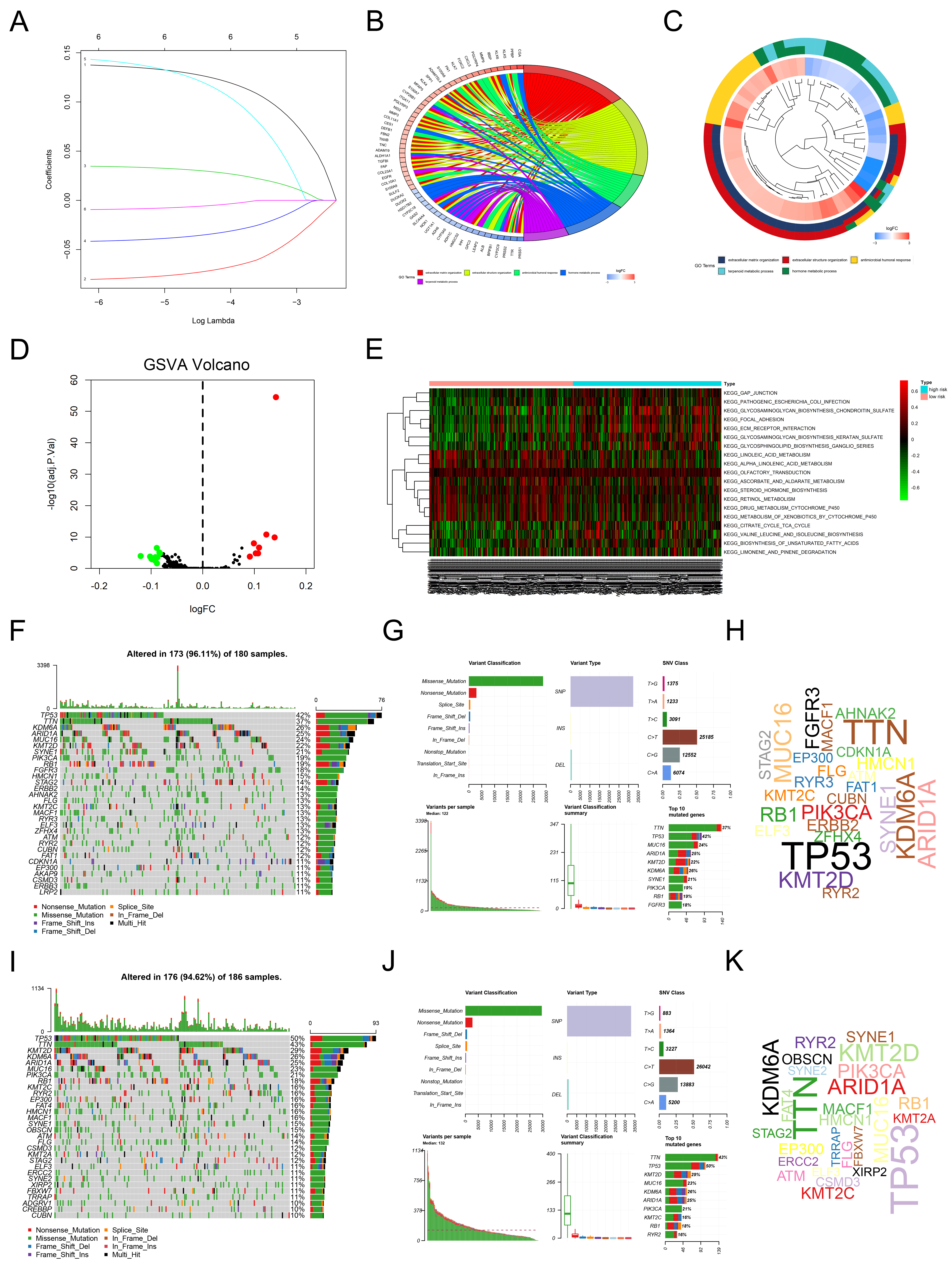


Figure. S5 Characteristics of risk score pattern and tumor somatic mutation. (A) The most regularized and parsimonious risk score pattern was built by multivariate Cox regression using LASSO Cox regression analysis. (B-C) Functional annotation of the genes with differentially expressed gene between high risk and low risk score groups using GO terms of biological processes. (D-E) GSVA enrichment analysis showed the status of biological pathways between two risk score group. Red represented activated pathways and green represented inhibited pathways. (D) Volcano plot, (E) Heatmap. (F-H) Primary tumor somatic mutation in low risk group (F) The waterfall plot of tumor somatic mutation, (G) Variant classification and type of genetic alterations; (H) Gene cloud map showed the mutant genes. The size of gene names represented the number of patients mutated for each gene. (I-K) Primary tumor somatic mutation in high risk group (I) The waterfall plot of tumor somatic mutation; (J) Variant classification and type of genetic alterations; (K) Gene cloud map showed the mutant genes. The size of gene names represented the number of patients mutated for each gene.


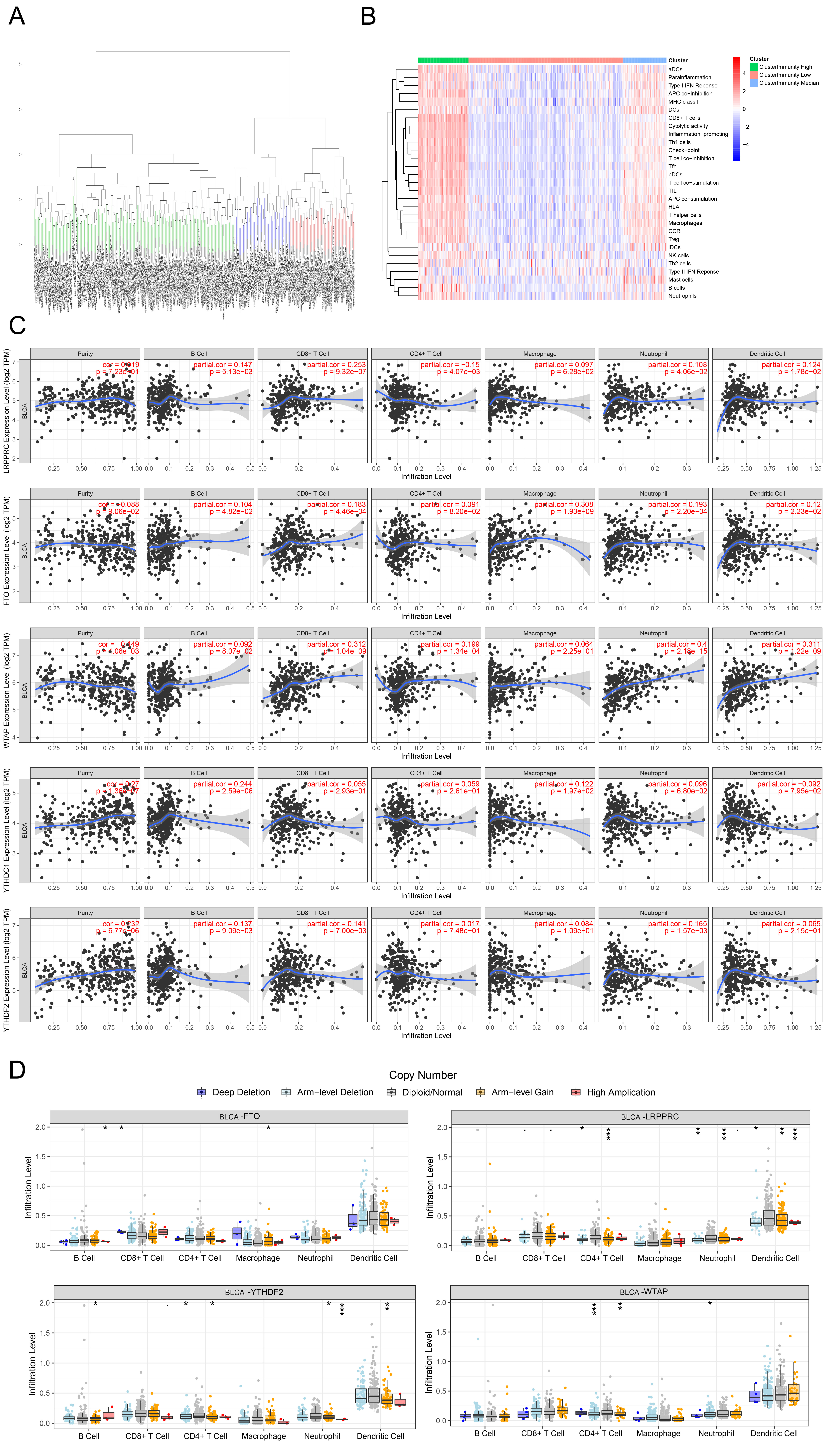


Figure. S6 Characteristics of immune signature with m6A regulators and risk score pattern. (A) The single-sample gene-set enrichment analysis (ssGSEA) showed the three distinct immunity clusters. (B) Heatmap showed the expression levels of 29 immune signatures in three distinct immunity clusters. Red represented high expression of regulators and blue represented low expression. (C) The association of m6A expression level with 6 immune cells and tumor purity. The data was obtained from TIMER website (https://cistrome.shinyapps.io/timer/). (D) The association of m6A regulators mutations with 6 immune cells. The data was obtained from TIMER website (https://cistrome.shinyapps.io/timer/). *P < 0.05, **P < 0.01, ***P < 0.001.


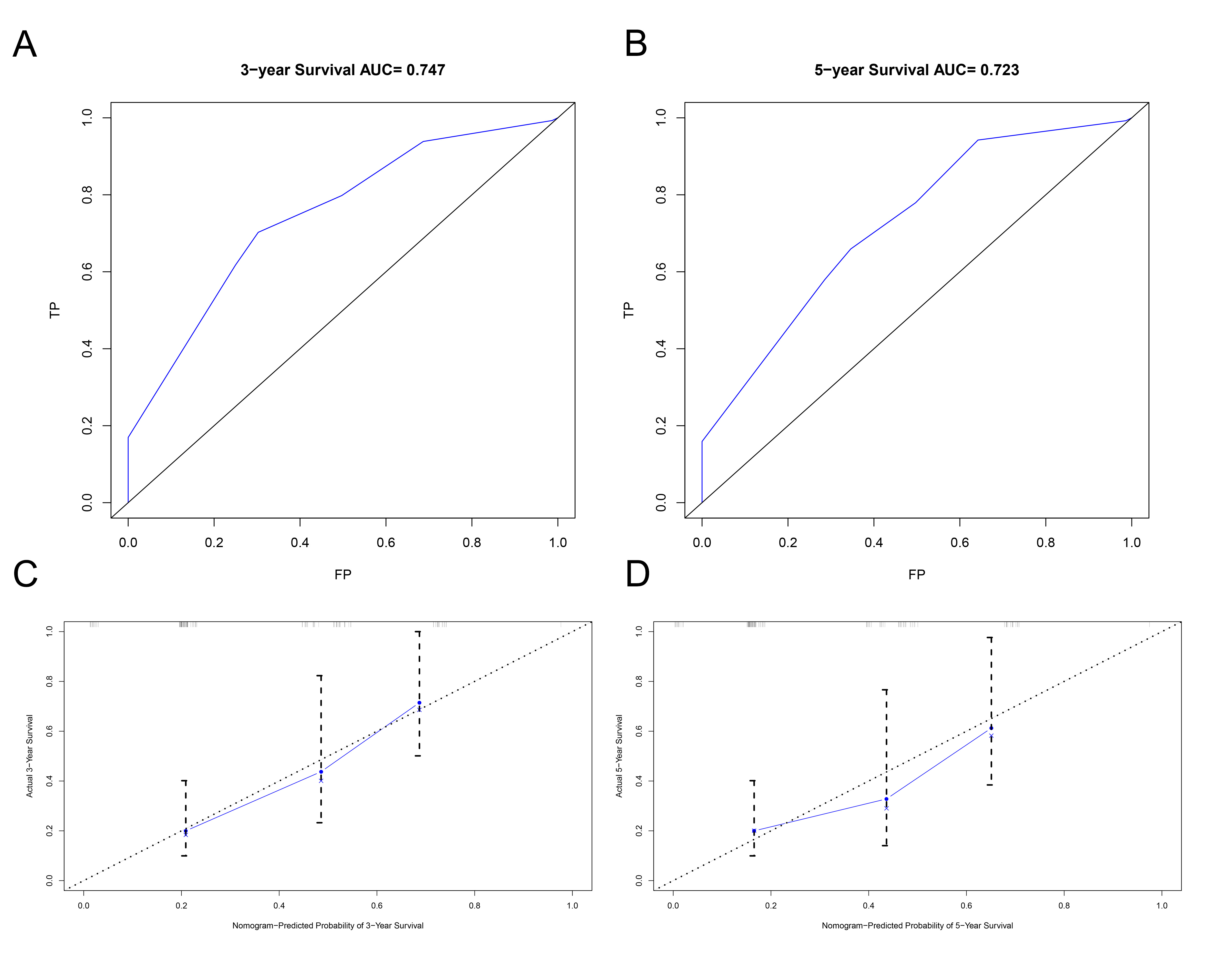


Figure. S7 Internal validation of nomogram predicting prognosis of individual patients (A-B) Receiver operating characteristic (ROC) curves of the nomogram for predicting (A) 3- and (B) 5-year overall survival (OS) status in validation cohort. (C-D) The calibration plots for predicting OS of patients at 3- (C) and 5- years (D) with bar (95% confdence interval) in validation cohort.

Supplemental Table 1 The 29 immune signatures represented by 29 different gene libraries

| Immune signatures | Gene names |
| --- | --- |
| aDCs | CD83 LAMP3 CCL1 |
| APC co inhibition | C10orf54 CD274 LGALS9 PDCD1LG2 PVRL3 |
| APC co stimulation | CD40 CD58 CD70 ICOSLG SLAMF1 TNFSF14 TNFSF15 TNFSF18 TNFSF4 TNFSF8 TNFSF9 |
| B cells | BACH2 BANK1 BLK BTLA CD79A CD79B FCRL1 FCRL3 HVCN1 RALGPS2 |
| CCR | CCL16 TPO TGFBR2 CXCL2 CCL14 TGFBR3 IL11RA CCL11 IL4I1 IL33 CXCL12 CXCL10 BMPER BMP8A CXCL11 IL21R TNFRSF9 IL17B ILF2 CX3CR1 CCR8 TNFSF12 CSF3 TNFSF4 BMP3 CX3CL1 BMP5 CXCR2 TNFRSF10D BMP2 CXCL14 CCL28 CXCL3 BMP6 CCL21 CXCL9 CCL23 IL6 TNFRSF18 IL17RD IL17D IL27 CCL7 IL1R1 CXCR4 CXCR2P1 TGFB1I1 IFNGR1 IL9R IL1RAPL1 IL11 CSF1 IL20RA IL25 TNFRSF4 IL18 ILF3 CCL20 TNFRSF12A IL6ST CXCL13 IL12B TNFRSF8 IL6R BMPR2 IFNE IL1RAPL2 IL3RA BMP4 CCL24 TNFSF13B CCR4 IL2RA IL32 TNFRSF10C IL22RA1 BMPR1A CXCR5 CXCR3 IFNA8 IL17REL IFNB1 IFNAR1 TNFRSF1B CCL17 IFNL1 IL16 IL1RL1 ILK CCL25 ILDR2 CXCR1 IL36RN IL34 TGFB1 IFNG IL19 ILKAP BMP2K CCR10 ILDR1 EPO CCR7 IL17C IL23A CCR5 IL7 EPOR CCL13 IL2RG IL31RA TNFAIP6 IFNL2 BMP1 IL12RB1 TNFAIP8 IL4R TNFRSF6B TNFAIP8L1 TNFRSF10B IFNL3 CCL5 CXCL6 CXCL1 CCR3 TNFSF11 CSF1R IL21 IL1RAP IL12RB2 CCL1 IL17RA CCR1 IL1RN TNFRSF11B TNFRSF14 IL13 IL2RB BMP8B CCL2 IL24 IL18RAP TGFBI TNFSF1 TNFRSF11A CXCL5 IL5RA TNFSF9 IL1RL2 TNFRSF13C IL36G IL15RA TNFRSF21 CXCL8 IL22RA2 TNFAIP8L2 IL18R1 IFNLR1 CXCR6 CCL3L3 TNFRSF1A IL17RE IFNGR2 IL17RC TNFAIP8L3 ILVBL TGFBRAP1 CCL4L1 CSF2RA CCRN4L CCL26 TNFAIP1 CCRL2 IFNA10 TNFRSF17 IFNA13 IL20 IL18BP CCL3L1 TNFSF12-TNFSF13 IL5 IL23R IL26 TNF TGFA CSF2 IL1F10 CXCL17 TNFSF13 IFNA4 IL37 IL12A IL7R IFNA1 IL1A IL4 IL2 CCL22 CSF3R IL10 IFNK TGFB2 IL1R2 IL1B IL17F IL27RA IL15 TNFSF8 IL36B XCL1 CXCL16 TNFRSF19 IL3 CCL3 IFNA2 BMPR1B IFNA21 TNFSF18 CCL8 IL17RB TNFRSF25 IL22 IL10RB IFNAR2 CCL18 IFNA16 CSF2RB IL36A TNFAIP3 IL13RA2 IL13RA1 CCR9 TNFRSF10A IFNA7 IFNW1 XCL2 TNFSF14 CCR2 BMP15 BMP10 CCL15 CCL14 TGFBR1 IFNA5 BMP7 IFNA14 IL20RB IL10RA IFNA17 CCR6 TGFB3 CCL15 CCL4 CCL27 TNFRSF13B TNFAIP2 IL31 IL17A TNFSF15 CCL19 IFNA6 IL9 |
| CD8+ T cells | CD8A |
| Check-point | IDO1 LAG3 CTLA4 TNFRSF9 ICOS CD80 PDCD1LG2 TIGIT CD70 TNFSF9 ICOSLG KIR3DL1 CD86 PDCD1 LAIR1 TNFRSF8 TNFSF15 TNFRSF14 IDO2 CD276 CD40 TNFRSF4 TNFSF14 HHLA2 CD244 CD274 HAVCR2 CD27 BTLA LGALS9 TMIGD2 CD28 CD48 TNFRSF2 CD40LG ADORA2A VTCN1 CD160 CD44 TNFSF18 TNFRSF18 BTNL2 C10orf54 CD200R1 TNFSF4 CD200 NRP1 |
| Cytolytic activity | PRF1 GZMA |
| DCs | CCL17 CCL22 CD209 CCL13 |
| HLA | HLA-E HLA-DPB2 HLA-C HLA-J HLA-DQB1 HLA-DQB2 HLA-DQA2 HLA-DQA1 HLA-A HLA-DMA HLA-DOB HLA-DRB1 HLA-H HLA-B HLA-DRB5 HLA-DOA HLA-DPB1 HLA-DRA HLA-DRB6 HLA-L HLA-F HLA-G HLA-DMB HLA-DPA1 |
| iDCs | CD1A CD1E |
| Inflammation-promoting | CCL5 CD19 CD8B CXCL10 CXCL13 CXCL9 GNLY GZMB IFNG IL12A IL12B IRF1 PRF1 STAT1 TBX21 |
| Macrophages | C11orf45 CD68 CLEC5A CYBB FUCA1 GPNMB HS3ST2 LGMN MMP9 TM4SF19 |
| Mast cells | CMA1 MS4A2 TPSAB1 |
| MHC class I | B2M HLA-A TAP1 |
| Neutrophils | EVI2B HSD17B11 KDM6B MEGF9 MNDA NLRP12 PADI4 SELL TRANK1 VNN3 |
| NK cells | KLRC1 KLRF1 |
| Parainflammation | CXCL10 PLAT CCND1 LGMN PLAUR AIM2 MMP7 ICAM1 MX2 CXCL9 ANXA1 TLR2 PLA2G2D ITGA2 MX1 HMOX1 CD276 TIRAP IL33 PTGES TNFRSF12A SCARB1 CD14 BLNK IFIT3 RETNLB IFIT2 ISG15 OAS2 REL OAS3 CD44 PPARG BST2 OAS1 NOX1 PLA2G2A IFIT1 IFITM3 IL1RN |
| pDCs | CLEC4C CXCR3 GZMB IL3RA IRF7 IRF8 LILRA4 PHEX PLD4 PTCRA |
| T cell co-inhibition | BTLA C10orf54 CD160 CD244 CD274 CTLA4 HAVCR2 LAG3 LAIR1 TIGIT |
| T cell co-stimulation | CD2 CD226 CD27 CD28 CD40LG ICOS SLAMF1 TNFRSF18 TNFRSF25 TNFRSF4 TNFRSF8 TNFRSF9 TNFSF14 |
| T helper cells | CD4 |
| Tfh | PDCD1 CXCL13 CXCR5 |
| Th1 cells | IFNG TBX21 CTLA4 STAT4 CD38 IL12RB2 LTA CSF2 |
| Th2 cells | PMCH LAIR2 SMAD2 CXCR6 GATA3 IL26 |
| TIL | ITM2C CD38 THEMIS2 GLYR1 ICOS F5 TIGIT KLRD1 IRF4 PRKCQ FCRL5 SIRPG LPXN IL2RG CCL5 LCK TRAF3IP3 CD86 MAL LILRB1 DOK2 CD6 PAG1 LAX1 PLEK PIK3CD SLAMF1 XCL1 GPR171 XCL2 TBX21 CD2 CD53 KLHL6 SLAMF6 CD40 SIT1 TNFRSF4 CD79A CD247 LCP2 CD3D CD27 SH2D1A FYB ARHGAP30 ACAP1 CST7 CD3G IL2RB CD3E FCRL3 CORO1A ITK TCL1A CYBB CSF2RB IKZF1 NCF4 DOCK2 CCR2 PTPRC PLAC8 NCKAP1L IL7R CD28 STAT4 CD8A LY9 CD48 HCST PTPRCAP SASH3 ARHGAP25 LAT TRAT1 IL10RA PAX5 CCR7 DOCK11 PARVG SPNS1 CD52 HCLS1 ARHGAP9 GIMAP6 PRKCB MS4A1 GPR18 TBC1D10C GVINP1 P2RY8 EVI2B VAMP5 KLRK1 SELL MPEG1 MS4A6A ARHGAP15 MFNG GZMK SELPLG TARP GIMAP7 FAM65B INPP5D ITGA4 MZB1 GPSM3 STK10 CLEC2D IL16 NLRC3 GIMAP5 GIMAP4 IFFO1 CFH PVRIG CFHR1 |
| Treg | IL12RB2 TMPRSS6 CTSC LAPTM4B TFRC RNF145 NETO2 ADAT2 CHST2 CTLA4 NFE2L3 LIMA1 IL1R2 ICOS HSDL2 HTATIP2 FKBP1A TIGIT CCR8 LTA SLC35F2 IL21R AHCYL1 SOCS2 ETV7 BCL2L1 RRAGB ACSL4 CHRNA6 BATF LAX1 ADPRH TNFRSF4 ANKRD10 CD274 CASP1 LY75 NPTN SSTR3 GRSF1 CSF2RB TMEM184C NDFIP2 ZBTB38 ERI1 TRAF3 NAB1 HS3ST3B1 LAYN JAK1 VDR LEPROT GCNT1 PTPRJ IKZF2 CSF1 ENTPD1 TNFRSF18 METTL7A KSR1 SSH1 CADM1 IL1R1 ACP5 CHST7 THADA CD177 NFAT5 ZNF282 MAGEH1 |
| Type I IFN Reponse | DDX4 IFIT1 IFIT2 IFIT3 IRF7 ISG20 MX1 MX2 RSAD2 TNFSF10 |
| Type II IFN Reponse | GPR146 SELP AHR |

Supplemental Table 2 RT-qPCR primers sequences

| Gene name | Primer | Sequences |
| --- | --- | --- |
| PD-L1 | Forward | TGGCATTTGCTGAACGCATTT |
|  | Reverse | TGCAGCCAGGTCTAATTGTTTT |
| IGF2BP3 | Forward | TATATCGGAAACCTCAGCGAGA |
|  | Reverse | GGACCGAGTGCTCAACTTCT |
| GAPDH | Forward | GGAGCGAGATCCCTCCAAAAT |
|  | Reverse | GGCTGTTGTCATACTTCTCATGG |

Supplemental Table 3 The m^6^A regulators with their coefficients from the LASSO analysis

| m^6^A regulators | Coefficient |
| --- | --- |
| IGF2BP3 | 0.13482125 |
| YTHDC1 | -0.078382878 |
| LRPPRC | 0.033549768 |
| WTAP | -0.040049438 |
| FTO | 0.137689017 |
| YTHDF2 | -0.008512461 |

Supplemental Table 4 Clinicopathological characteristics stratified by different risk score groups

|  |  | Risk Score | |  |
| --- | --- | --- | --- | --- |
| Variables | Total patients | Low | High | p value |
| Age |  |  |  | 0.433 |
| ≤65 | 139 | 72 | 67 |  |
| >65 | 227 | 108 | 119 |  |
| Gender |  |  |  | 0.255 |
| Female | 93 | 41 | 52 |  |
| Male | 273 | 139 | 134 |  |
| Grade |  |  |  | 0.004 |
| Low | 19 | 16 | 3 |  |
| High | 346 | 163 | 183 |  |
| Unknown | 1 | 1 | 0 |  |
| T |  |  |  | 0.023 |
| T0 | 1 | 1 | 0 |  |
| T1 | 3 | 3 | 0 |  |
| T2 | 115 | 66 | 49 |  |
| T3 | 190 | 80 | 110 |  |
| T4 | 57 | 30 | 27 |  |
| N |  |  |  | 0.036 |
| N0 | 218 | 113 | 105 |  |
| Nx | 26 | 18 | 8 |  |
| N1 | 43 | 17 | 26 |  |
| N2 | 73 | 31 | 42 |  |
| N3 | 6 | 1 | 5 |  |
| M |  |  |  | 0.041 |
| M0 | 177 | 98 | 79 |  |
| Mx | 181 | 80 | 101 |  |
| M1 | 8 | 2 | 6 |  |
|  |  |  |  |  |
|  |  |  |  |  |
|  |  |  |  |  |
|  |  |  |  |  |
|  |  |  |  |  |
